# Microfluidic biosensors for biomarker detection in body fluids: a key approach for early cancer diagnosis

**DOI:** 10.1186/s40364-024-00697-4

**Published:** 2024-12-05

**Authors:** Zhiting Liu, Yingyu Zhou, Jia Lu, Ting Gong, Elena Ibáñez, Alejandro Cifuentes, Weihong Lu

**Affiliations:** 1 School of Medicine and Health, Harbin Institute of Technology, 92 Xidazhi Street, Nangang District, Harbin, 150001 China; 2 School of Mechatronics Engineering, Harbin Institute of Technology, 92 Xidazhi Street, Nangang District, Harbin, 150001 China; 3 Zhengzhou Research Institute, Harbin Institute of Technology, Zhengzhou, Henan China; 4 National and Local Joint Engineering Laboratory for Synthesis Transformation and Separation of Extreme Environmental Nutrients, 92 Xidazhi Street, Nangang District, Harbin, 150001 China; 5 Laboratory of Foodomics, Institute of Food Science Research, CIAL, CSIC, Nicolás Cabrera 9, Madrid, 28049 Spain

**Keywords:** Microfluidics, Early cancer detection, Biomarkers, Oncology, Hematology, Personalized nanomedicine

## Abstract

Early detection of cancer significantly improves patient outcomes, with biomarkers offering a promising avenue for earlier and more precise diagnoses. Microfluidic biosensors have emerged as a powerful tool for detecting these biomarkers in body fluids, providing enhanced sensitivity, specificity, and rapid analysis. This review focuses on recent advances in microfluidic biosensors from 2018 to 2024, detailing their operational principles, fabrication techniques, and integration with nanotechnology for cancer biomarker detection. Additionally, we have reviewed recent innovations in several aspects of microfluidic biosensors, such as novel detection technologies, nanomaterials and novel microfluidic chip structures, which significantly enhance detection capabilities. We highlight key biomarkers pertinent to early cancer detection and explore how these innovations in biosensor technology contribute to the evolving landscape of personalized medicine. We further explore how these technologies could be incorporated into clinical cancer diagnostic workflows to improve early detection and treatment outcomes. These innovations could help enable more precise and personalized cancer diagnostics. In addition, this review addresses several important issues such as enhancing the scalability and sensitivity of these biosensors in clinical settings and points out future possibilities of combining artificial intelligence diagnostics with microfluidic biosensors to optimize their practical applications. This overview aims to guide future research and clinical applications by addressing current challenges and identifying opportunities for further development in the field of biomarker research.

## Introduction

Cancer incidence and mortality rates keep increasing yearly [1]. The complexity of curing cancer partially arises from the inconspicuous nature of early symptoms that usually results in delayed diagnoses and treatments [2, 3]. Previous studies have shown the pivotal role of regular breast cancer screening in reducing mortality rates by approximately 40% for women [4]. Similarly, early screening for lung cancer has demonstrated a 20% reduction in mortality [5], while early detection and treatment can reduce mortality rates for squamous cell carcinoma of the esophagus by 30–60% [6]. Prostate cancer, when subject to early screening through Prostate-specific Antigen (PSA), exhibits a mortality reduction of 21% [7]. Early cancer diagnosis serves as a critical intervention that impedes malignant tumor progression and mitigates cancer fatality.

Pathological diagnosis is regarded as the gold standard for cancer diagnosis [8]. Current diagnostic methodologies employ scanning and imaging techniques such as X-ray scanning, magnetic resonance imaging (MRI), ultrasound testing, and gastrointestinal scintigraphy [9]. Diagnosis often relies on the imaging and evaluation of visible cancer lesions at the scrutinized sites [10–14]. Additionally, liquid biopsy, which detects cancer biomarkers at the nanoscale in body fluids using techniques such as polymerase chain reaction (PCR), mass spectrometry, chromatography and electrophoresis, has also facilitated cancer detection [15]. However, the intricate sample pre-treatment and specialized operational requirements often make liquid biopsy time-consuming and cost-intensive [16]. Despite its promise, liquid biopsy could fail to detect low-concentration cancer nano biomarkers in early-stage cancer.

To address these issues, researchers have tried to enhance liquid biopsy sensitivity and maneuverability by integrating it with other methods [17]. Among these innovations, microfluidic technology stands out with markedly improved sensitivity and selectivity in diagnosis [18, 19]. Microfluidic devices consist of multiple micro-elements and can manipulate liquid droplets at nano- or micro-meter scale, which provides advantages such as compact size, portability, minimal sample consumption, shortened processing time, increased maneuverability, and improved sensitivity. These features make microfluidic devices promising for more efficient, timely, and on-site early cancer diagnoses.

Another key advancement of microfluidic devices in the field of cancer detection is the integration of microfluidics with novel detection technologies, resulting in various detection devices with distinct characteristics. For example, electrochemical sensors have high sensitivity, making microfluidic devices incorporating electrochemical sensors suitable for detecting low-concentration biomarkers [20]. Fluorescence technology has also made significant progress in cancer biomarker detection by utilizing fluorescent labeling to achieve high specificity. This makes microfluidic devices with fluorescent labeling suitable for precise diagnostics even at very low biomarker concentrations, which is crucial for early detection [21]. Additionally, the development of nanoparticles has expanded the detection limits of various markers and enables the capture of rare biomarkers [22]. Surface Enhanced Raman Scattering (SERS) relies on the interaction between nanoparticles and biomarkers to dramatically amplify the Raman signal, leading to much improved detection capabilities [23]. Each of these integrated approaches significantly improves the performance of microfluidic devices in early cancer detection.

Given the enormous potential of biosensors in liquid biopsy, their development in healthcare is particularly promising. Microfluidic biosensors can achieve precise biomarker detection by utilizing advanced techniques such as affinity binding, where specific antibodies or molecular probes selectively capture target biomarkers from complex biological samples including blood or urine. By accurately identifying these biomarkers, clinicians can tailor diagnostic information and treatment plans that fit each patient’s specific tumor characteristics, leading to more effective and targeted therapies. For instance, microfluidic biosensors can detect circulating tumor DNA (ctDNA) or specific proteins released by tumors, providing insight into the tumor’s genetic mutations and biochemical environment. Such information helps in decision making of the most appropriate treatment options, including targeted therapies that directly address the identified mutations or characteristics [24]. Innovations in nanotechnology and microfluidic systems are pushing biosensor development towards greater miniaturization and integration. This enables real-time monitoring of individual health conditions while offering strong support for telemedicine and mobile health services [25].

Moreover, the introduction of new functional materials, such as nanoparticles, quantum dots, and graphene, has significantly enhanced the sensitivity, selectivity, and stability of microfluidic biosensors. For instance, graphene’s exceptional conductivity and mechanical strength could help improve the sensitivity of biosensors, enabling the detection of trace cancer biomarkers with greater precision [26]. Similarly, quantum dots (QDs) offer unique optical properties, including size-tunable fluorescence and high photostability, which enhance the sensitivity and specificity of biomarker detection. Their ability to emit light at specific wavelengths allows for multiplexing, enabling simultaneous detection of multiple biomarkers in a single assay [27]. Additionally, some special nanoparticles, due to their high surface area and unique optical properties, can enhance selectivity by facilitating specific interactions with target molecules [28]. These materials stimulate innovation in design, greatly broadening the application areas of microfluidic biosensors [29]. It is worth noting that the recent rapid development of artificial intelligence (AI) and machine learning (ML) presents exciting opportunities for enhancing these biosensors. For instance, AI algorithms can analyze data from microfluidic biosensors in real time, improving the promptness of biomarker detection [30]. ML models can identify complex patterns in biological signals that traditional methods might overlook, leading to more accurate cancer diagnoses [31]. If artificial intelligence and machine learning technology can be deeply integrated into microfluidic chips, it should greatly enhance the data analysis capabilities of microfluidic biosensors, enabling them to efficiently process complex biological signals and make more accurate predictions, which is expected to further consolidate the position of biosensors in medical diagnosis.

Despite the promising potential of microfluidic biosensor technology, several significant challenges hinder its wider adoption. One major issue is long-term stability: many biosensors degrade over time or under varying environmental conditions, thus compromising their reliability. Cost control is another critical factor, as high expenses associated with advanced materials and fabrication techniques can limit technology accessibility. Developing cost-effective manufacturing methods is essential to overcome this obstacle. Additionally, evolving regulatory policies can create uncertainties for manufacturers, slowing down the introduction of innovative products. Collaborative efforts among researchers, regulatory bodies, and industry stakeholders are necessary to establish clear regulations that ensure safety while not compromising innovation. With ongoing technological advancement and market maturity, it’s anticipated that these challenges could be overcome.

In summary, the combination of biosensor technology and microfluidics is expected to become an indispensable tool in the diagnosis, treatment, and prevention of diseases, opening up a new era for the healthcare field. In nanomedical research, microfluidic biosensor devices show promise in a variety of applications [32, 33]. This review provides a comprehensive overview of microfluidic systems and their applications in early cancer detection mechanisms. It also outlines the fabrication methods of these microfluidic devices and highlights some promising biomarkers. Recent scientific achievements in early cancer detection and advances in the fabrication of microfluidic biosensors are summarized. This review combines these aforementioned elements into a single article, aiming to provide engineering and medical researchers with a scientific and comprehensive guide. Additionally, the integration of artificial intelligence with microfluidic biosensors is emphasized, showing its potential in improving diagnostic accuracy and promoting the development of personalized medicine. To expedite the contribution of nano-microfluidics biosensor to early cancer screening for the whole population, the review suggests addressing some urgent issues and explores promising future directions for microfluidic biosensor in nanomedicine, thereby contributing to broader healthcare research.

## Principles and fabrication technologies of microfluidic biosensors

### Principles and characteristics of microfluidic devices

**Fig. 1:**
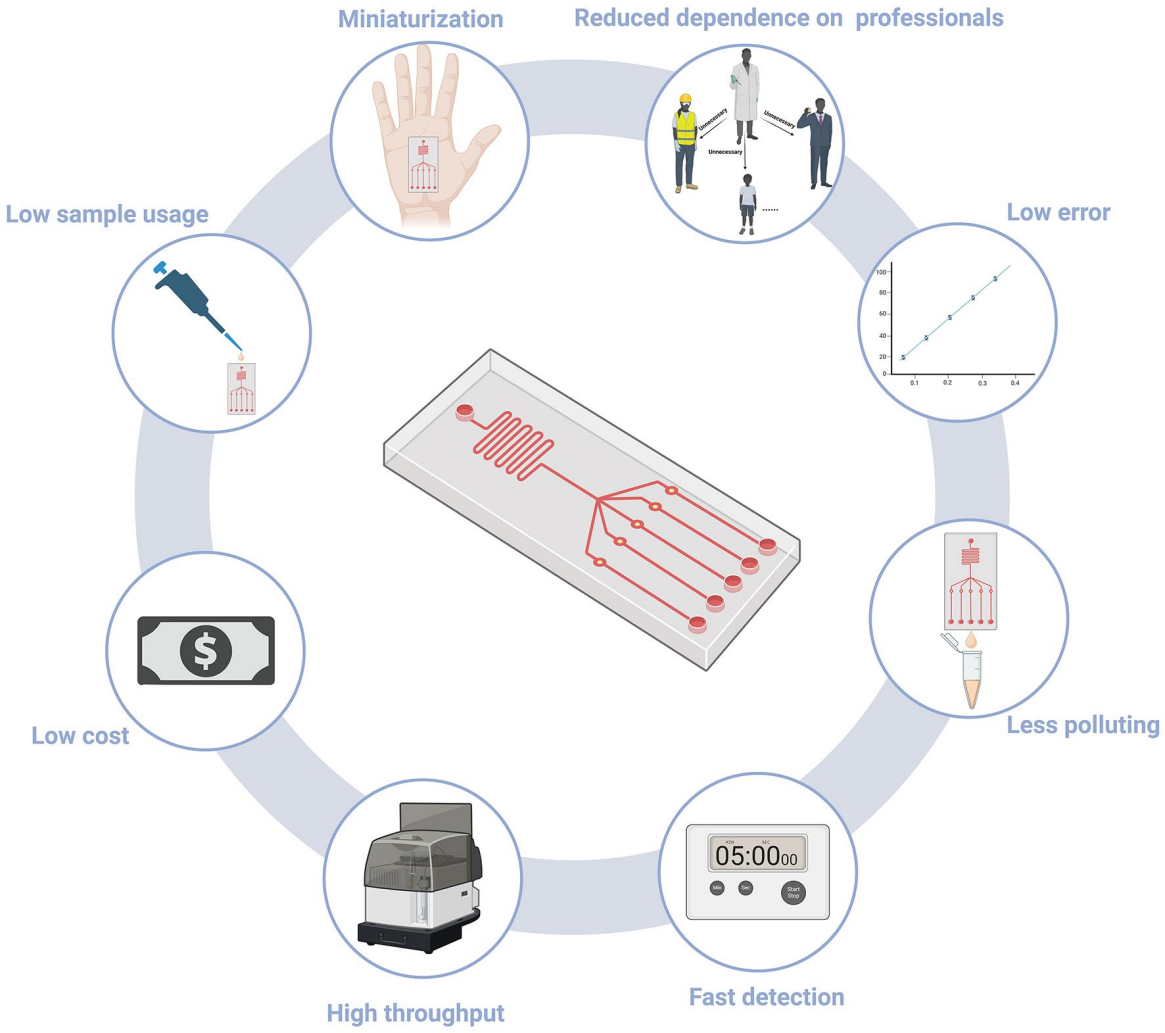
Advantages of microfluidic biosensors include low error, low sample usage, miniaturization, reduced dependence on professionals, less pollution, fast detection, high throughput, and low cost

To gain a comprehensive understanding of the profound advantages offered by microfluidic biosensors in early cancer detection, one must first acquire knowledge regarding the fundamental principles and distinctive characteristics of microfluidic devices. Microfluidics, the manipulation of minute fluids within microchannels, is commonly referred to as lab-on-a-chip or micro total analytical systems [34]. In the biomedical field, researchers often integrate nanomaterials, antibodies, or various probes into designated ‘workshops’ within a microfluidic device, forming diverse types of microfluidic biosensors. This integration facilitates the precise separation of specific substances, thereby enhancing the sensitivity and selectivity of these microsystems [35]. As shown in Fig. 1, the miniaturization of microfluidic biosensors leads to low sample usage and fast detection, enhancing portability and reducing costs. This unique design minimizes error rates and environmental impact while enabling high-throughput analyses. Furthermore, these devices require less specialized training, making them accessible for broader use in various settings [36]. These characteristics collectively make microfluidic biosensors a transformative tool, rendering early diagnosis of cancer and other diseases more efficient, timely, and amenable to on-site applications.

A key factor in the rapid development of microfluidic systems in the biomedical field has been the integration of nanomaterials, which further improves biosensor sensitivity and selectivity. The integration of microfluidic systems with nanomaterials significantly enhances sensing parameters by providing high surface-to-volume ratios and facilitating efficient molecular interactions [37]. Microfluidic devices enable precise fluid control and manipulation at the microscale, promoting rapid mixing and reaction of target analytes with sensing elements. This leads to improved sensitivity and selectivity of biosensors. Additionally, the miniaturization inherent in microfluidic technology reduces sample and reagent consumption, shortens detection time, and allows for portable and on-site diagnostic applications. The incorporation of nanomaterials, such as gold nanoparticles (AuNPs), carbon nanotubes (CNTs) and quantum dots (QDs) into microfluidic systems further amplifies the detection signals due to their unique physical and chemical properties. For example, AuNPs could enhance electrochemical and optical signals; their excellent conductivity improves electron transfer in electrochemical sensors, lowering detection limits. They can also serve as substrates for biomolecules attachment, increasing the density of active sites for molecular binding and improving signal amplification. CNTs contribute to enhanced stability and faster electron transfer rates particularly in electrochemical applications due to their high electrical conductivity and large surface area, which enable efficient capture and transduction of signals from low-concentration biomarkers. Graphene, with its two-dimensional structure, offers a high surface area and excellent conductivity, making it ideal for enhancing the sensitivity of both optical and electrochemical sensors. Additionally, its mechanical flexibility is advantageous in the development of flexible and wearable biosensors. QDs, semiconductor nanocrystals known for their size-tunable fluorescence, are utilized in microfluidic biosensors for highly sensitive optical detection, allowing for accurate identification of low-concentration biomarkers [26, 27, 38].

However, there are also challenges in integrating these nanomaterials into microfluidic devices for large-scale production of microfluidic biosensors, including ensuring uniform distribution and effective bonding of nanomaterials to maintain consistent performance. Furthermore, scaling up these technologies for mass production poses additional challenges, such as achieving reproducibility and minimizing production costs. Optimizing synthesis methods and establishing standardized production plants for nanomaterial integration can help improve consistency and efficiency. Addressing these challenges is crucial to fully realizing the potential of microfluidic biosensors, especially for early diagnosis of cancer and other diseases.

This synergy between microfluidics and nanomaterials transforms biosensing, making it possible to detect low-abundance biomarkers with high accuracy and reliability.

### Fabrication technologies and materials for microfluidic biosensors

The advancement of microfluidic biosensors can be largely attributed to micro-fabrication technologies for making microfluidic chips [39–42]. Such technologies include photolithography, injection molding, thermocompression, laser ablation, 3D printing and so on.

Photolithography has played an essential role in microfluidic chip fabrication since the 1990s. It is widely used for creating micron-sized structures on silicon or glass substrates due to its high accuracy. The photolithography process involves several steps: first, the substrate surface is cleaned and coated with a layer of photoresist. Next, the desired pattern is transferred to the photoresist by either UV exposure through a mask or direct laser writing without any mask. After development, the photoresist is selectively removed from either the exposed or unexposed areas depending on the type of photoresist used, defining the channel regions. Finally, wet or dry etching removes the corresponding material layer in the substrate, forming the microfluidic channels. Despite its accuracy, photolithography’s productivity is limited due to its multi-step nature, reliance on specialized equipment, and the need for a cleanroom environment [43–45].

In response to the limitations of photolithography for mass production, injection molding was introduced in the early 2000s. This process offers significantly higher throughput and is particularly suitable for producing large quantities of polymer-based microfluidic chips. Injection molding involves fabricating a mold from metal or silicon, heating a thermoplastic polymer such as cyclic olefin copolymer (COC) until it is molten, injecting it into the mold under high pressure, and releasing the solidified chip after cooling. While injection molding greatly reduces production time, the high upfront costs and time required for mold fabrication make it less suitable for rapid prototyping. Hot embossing emerged as an alternative for low-volume production and rapid prototyping in the late 1990s. In this process, a prefabricated mold is imprinted onto a polymer substrate under controlled temperature and pressure to create microchannel patterns. Unlike injection molding, thermal embossing does not require the polymer to reach a fully molten state, which simplifies temperature control. This technique is suited for medium-volume production but is still limited by long cycle times and the need for precise control. Additionally, mold wear and consistency in replication can pose challenges [46, 47]. Laser ablation became popularized in the early 21st century as microfluidic chip design required greater flexibility and precision. This technique uses high-energy laser pulses to precisely ablate microchannels and other structures on substrates such as polymers, metals, or glass. The process includes cleaning the substrate, laser patterning, and subsequent cleaning and surface treatment to ensure integrity. Laser ablation is highly accurate and compatible with various materials, making it ideal for small batch production and prototyping. However, its relatively slow processing speed limits its application in large-scale production [48].

In recent years, 3D printing has emerged as a promising technique for microfluidic device fabrication. Also known as additive manufacturing, this method is based on the layer-by-layer printing and forming of devices, with advantages such as plentiful candidate printing materials, the ability to create intricate structures without molds, and rapid and customizable prototyping. Traditional extrusion-based 3D printing (e.g., fused deposition modeling, FDM) has a resolution of about 100–1000 micrometers, which limits its usage in highly precise microfluidic devices. For applications requiring micro-scale features, photopolymerization-based 3D printing methods such as stereolithography appearance (SLA) and digital light processing (DLP) are commonly employed. These techniques can achieve resolutions down to tens of micrometers, making them suitable for building intricate microfluidic structures. The nascent application of 3D printing in microfluidic chip fabrication is promising in a much broader field in the future [42, 49, 50]. Table 1 provides an overview of the various techniques employed in microfluidic device fabrication.

As the two most used and promising methods for the future, this paper focuses on soft lithography and 3D printing. Soft lithography and 3D printing each possesses unique advantages and potential in the fabrication of microfluidic biosensors. Soft lithography is a nanofabrication technique based on elastomeric molds, typically using polydimethylsiloxane (PDMS) and other elastomeric materials. The fundamental process involves creating microstructured templates on silicon wafers via traditional photolithography, then replicating these templates with elastomeric materials to form soft molds bearing microstructures. Subsequently, the soft mold is aligned with the target substrate and pressed, transferring the microstructures, followed by chemical or physical treatment of the substrate material to form the required microfluidic channels or devices [51]. At the same time, soft lithography also has certain disadvantages. For example, the process is inherently complex, involving multiple steps such as photolithography, etching and molding, each requiring precise control. This complexity is due to the necessity for strict conditions and precise operations at each step to avoid structural deformation or defects. Additionally, there are limitations in material compatibility, as some materials may not be suitable for mold fabrication and transfer processes, thus restricting its application scope. Furthermore, the multi-step nature of the process results in longer preparation time, impacting production efficiency [52]. Recent advancements in soft lithography have overcome several traditional limitations, such as time-consuming processes and limited material selection. For instance, rapid prototyping techniques incorporating laser-based fabrication methods enable faster mold creation, significantly reducing time cost and process complexity compared to traditional photolithographic ones. Concurrently, advances in materials science have led to the development of versatile elastomers and hybrid polymers, expanding the range of process-compatible materials and enabling the creation of softer and stretchable microfluidic systems suitable for wearable device applications. Additionally, techniques such as nanoimprinting have enhanced the precision of pattern transfer, achieved higher resolution and reduced defects in microstructures. Collectively, these innovations enhance the adaptability of soft lithography across various scales and configurations, broadening its application in complex microfluidic systems and improving overall production efficiency [53].

In comparison, 3D printing technology exhibits greater potential for future microfluidic chip fabrication. One of its primary advantages is the ability to directly fabricate complex microstructures without the need for molds, which makes design and prototyping more flexible and rapid. This capability is particularly beneficial for developing intricate and custom designs that are difficult to achieve with conventional methods. The suitability of 3D printing for various materials, including photosensitive resins, thermoplastics, and biomaterials, provides additional flexibility for microfluidic chip design and applications. A notable advantage is the rapid prototyping capability, crucial for the iterative design and testing of microfluidic chips. Compared to traditional manufacturing techniques, 3D printing significantly shortens development cycles and reduces costs, thereby facilitating faster innovation and deployment [42].

However, 3D printing also faces several challenges. The current resolution of 3D printing technology is not yet comparable to that of soft lithography, limiting its use in nanoscale structure fabrication. To address this issue, researchers have developed high-resolution techniques such as two-photon polymerization (TPP). TPP uses femtosecond laser pulses to initiate polymerization at the focal point, allowing for feature sizes down to the nanometer scale, thus significantly improving the precision of printed microstructures [54]. Another promising approach is nano-jet printing, which allows for the continuous deposition of materials at nanoscale dimensions, increasing the resolution and accuracy [55]. These advancements make it possible to achieve resolutions approaching those of traditional photolithographic methods.

Overall, soft lithography and 3D printing technologies have their unique advantages and application scenarios in the manufacturing of microfluidic devices. In contrast, 3D printing technology, due to its design flexibility and rapid prototyping capability, will exhibit enormous potential in the development of microfluidic chips with complex structures. As these two technologies continue to develop, the future holds the promise of widespread applications and significant breakthroughs in microfluidic biosensors across biomedical, environmental monitoring, and other fields.

Currently, researchers are actively developing new elastomeric materials and improving mold fabrication processes to enhance manufacturing precision and material compatibility. For example, optimizing the chemical composition and hardness of PDMS or adopting multilayer mold techniques can significantly improve process performance and production efficiency. The ongoing research aims to refine these processes to ensure more reliable and versatile applications.

The selection of the right substrate material is also important in processing microfluidic biosensors. Commonly employed materials include paper, polymer, silicon, and glass [56, 57]. Each material has unique advantages and disadvantages, making them suitable for different applications. Paper-based microfluidic chips are favored for point-of-care diagnostics, environmental monitoring, and food safety testing due to their low cost, simple manufacturing, portability, and capillary action, which negates the need for external pumps. However, their limitations include restricted chemical compatibility, low mechanical strength, and limited precision. Manufacturing techniques such as wax printing, inkjet printing, and laser cutting are suited for these applications [58]. In contrast, polymer-based microfluidic chips offer higher moldability, transparency, biocompatibility, and excellent elasticity, making them ideal for biomedical research, drug screening, cell culture, and complex fluid manipulation [59]. Despite challenges related to chemical and thermal stability and potential surface property changes, techniques like soft lithography and 3D printing effectively address these issues, enabling the creation of complex three-dimensional structures.

For applications requiring high precision and stability, silicon-based microfluidic chips are more suitable. Silicon’s ability to form high-precision microstructures and its ease of integration with electronic components make it indispensable in laboratory analysis, lab-on-a-chip systems, and high-performance detection devices [59]. Nonetheless, the high cost, brittleness, and complex fabrication processes of silicon-based chips limit their widespread use. Photolithography and wet etching techniques are particularly suitable for producing these high-precision microstructures.

Glass-based microfluidic chips, known for their chemical stability, optical transparency, high-temperature resistance, and ease of surface modification, are commonly used in high-sensitivity analyses, optical detection systems, and controlled chemical reactions. Surface modification is a critical factor in enhancing the performance of glass-based chips. For instance, silanization, which involves coating the glass surface with organosilane compounds, improves biocompatibility and enables the attachment of biomolecules, making these chips suitable for biosensing applications. Additionally, functional group coating (carboxyl or amino groups) can be introduced onto the glass surface to enhance its chemical reactivity, allowing for more selective and sensitive detection of target analytes. These modifications not only improve the chip’s interaction with biological samples but also significantly enhance its suitability for optical detection and high-sensitivity reactions. Although the high cost, brittleness, and complex fabrication processes of glass present challenges, their unique properties make them useful in analytical and detection applications. Suitable manufacturing techniques for glass-based chips include photolithography and laser machining [60].

In summary, the selection of materials for microfluidic biosensors, combined with appropriate fabrication techniques, enables the development of high-performance, low-cost, and user-friendly devices tailored to specific application requirements.

**Table 1:** Technologies for making microfluidic devices

Technology	Principle	Advantages	Disadvantages
Photolithography	Utilizes photosensitive resist to pattern a substrate	Offers high precision across a diverse range of materials	Requires specialized equipment and techniques
Injection molding	Involves heating raw material, injecting into a mold, and thermal curing	Repeatable, cost-effective, suitable for mass production	Involves a complex and technically demanding mold preparation process
Hot embossing	Processes polymer substrates using molds at appropriate temperatures and pressures	Simple operation, low cost, suitable for mass production of chips	Requires higher equipment and technology standards
Laser ablation	Selectively removes material with a laser to create microchannels and other features	High precision, flexible design, compatibility with a wide range of materials	Requires higher equipment and technology standards
3D printing	Involves direct layer-by-layer printing of devices using 3D printing technology	Wide material choice, ability to create complex structures, supports rapid prototyping and personalized design	Printing accuracy and speed are limited by the device

## Key biomarkers in cancer detection

Biomarkers have a prominent role in disease detection and are key elements in achieving highly sensitive and specific detection. Because of this, microfluidic devices have gained popularity in various types of nanomedicine diagnostics, such as novel coronaviruses detection [61], vascular risk analysis associated with type 2 diabetes [62], swift diagnosis of venereal pathogens [63], bacterial meningitis [64], and cancer diagnosis [65]. Current research suggests that early detection of cancer significantly reduces mortality rates. Liquid biopsies, being minimally invasive and rapid, can greatly facilitate early detection, while microfluidic biosensors streamline and enhance the efficiency of liquid biopsies [66]. Therefore, it is reasonable to believe that microfluidic biosensors can be more effective in reducing cancer mortality if they are applied in clinical settings. This efficacy arises from microfluidic devices’ inherent capability of autonomously achieving the separation and purification of target substances.

In the early stage of cancer diagnosis, the substances necessitating separation and identification typically revolve around cancer biomarkers. The sensitivity and specificity exhibited by microfluidic devices in biomarker separation are directly linked to the specific types of biomarkers under consideration. Therefore, the identification of biomarkers relevant to early cancer detection is imperative for a comprehensive understanding of the advantages offered by microfluidic biosensors. In cancer research, these biomarkers commonly originate from cancer cells themselves or represent products of the body’s immune response to cancer. The discernment of these biomarkers is indispensable to ascertaining the existence of cancer within the body and determining its stage.

Common cancer biomarkers include circulating tumor cells (CTCs) at microscale, as well as nano-biomarkers such as microRNAs (miRNAs), proteins, and small extracellular vesicles (sEVs) at nanoscale. Each category possesses unique diagnostic potential and contributes significantly to cancer diagnosis. During the development and progression of cancer, significant alterations occur in biomarkers present in body fluids that characterize tumor cell behavior and cancer progression. Upon tumorigenesis, cancer cells often secrete or overexpress specific soluble proteins and surface antigens, with elevated levels of these molecules frequently associated with enhanced proliferation and immune evasion mechanisms. miRNAs, key regulators of gene expression, frequently display dysregulation in cancers as characterized by aberrant up-regulation or down-regulation. These changes can disrupt cellular processes such as proliferation and apoptosis, ultimately promoting tumor growth and progression. Furthermore, during tissue invasion, CTCs are released from the primary tumor into the bloodstream, where their increasing abundance correlates with a higher likelihood of metastasis. Moreover, cell-free DNA (cfDNA) released during cell death, along with circulating tumor DNA (ctDNA) derived from tumor cells, show markedly elevated levels in cancer patients. Notably, ctDNA carries critical information on tumor-specific mutations, making it a valuable tool for non-invasive genetic testing. Exosomes, another type of nanoscale vesicle, are secreted in larger quantities by active tumor cells and serve as carriers of essential oncogenic signals, including proteins, RNA, and lipids [67]. The alterations in these biomarkers mirror critical oncogenic processes such as abnormal metabolism, unchecked proliferation, and immune evasion. These features make them indispensable for the early detection of cancer and for tracking disease progression.

### Secreted soluble proteins and surface antigens

The genetic mutation could induce transformation of normal tissue cells into cancer cells. As cancer cells proliferate or encounter immune system recognition and attack, they release distinctive nanoscale proteins into the bloodstream and other body fluids. These proteins, capable of directly or indirectly identifying cancer cells, are termed tumor-specific proteins. The extensively studied tumor-specific proteins are carcinoembryonic antigen (CEA) [68], alpha-fetoprotein (AFP) [69], PSA [70], chorionic gonadotropin (HCG) [71], carbohydrate antigen 19 − 9 (CA19-9) [72], carbohydrate antigen 12 − 5 (CA12-5) [73], carbohydrate antigen 72 − 4 (CA72-4) [74], carbohydrate antigen 24 − 2 (CA24-2) [75], carbohydrate antigen 15 − 3 (CA15-3) [76], cytokeratin 19 fragment (CYFRA21-1) [77], neuron-specific enolase (NSE) [78], and squamous cell carcinoma-associated antigen (SCC) [79]. In healthy people, these protein markers exhibit specific concentration ranges. Concentrations beyond these ranges indicate a potential cancer risk, necessitating further diagnostic evaluation. Most people are usually tested for the concentration of these proteins during blood tests, so it is crucial to know the normal concentration range of these proteins in the body. The concentration ranges of these proteins in a normal organism are summarized in Table 2, which is available to those who need it. Given the potential significance of these markers in early cancer diagnosis, researchers have recently engineered a variety of microfluidic devices specifically designed for the detection of these markers in body fluids [80–89].

**Table 2:** Protein markers for cancer diagnosis

Project	Normal physiological concentration range	Body fluid types	Cancer types
CEA	≤ 5.00 ug/L	Serum, cerebrospinal fluid, milk, gastric fluid, urine, feces	Colorectal cancer, breast cancer, lung cancer [68]
AEP	≤ 7.00 ug/L	Serum, urine, semen, feces	Liver cancer, pancreatic cancer, stomach cancer, lung cancer [69]
PSA	≤ 4.00 ug/L	Serum, semen	Prostate cancer [70]
HCG	Male ≤ 2.60 mIU/mL Healthy unfertilized women ≤ 5.30 mIU/mL Postmenopausal women ≤ 8.30 mIU/mL	Serum, urine, milk	Chorionic epithelial cell carcinoma, ovarian cancer [71]
CA 19 − 9	≤ 30.00 KU/L	Serum	Colorectal cancer, pancreatic cancer, Stomach cancer, liver cancer, lung cancer, ovarian cancer, breast cancer [72]
CA 125	Male ≤ 24.00 KU/L Women (18–49 years old) ≤ 47.00 KU/L Women (50 years old and above) ≤ 25.00 KU/L	Serum	Ovarian cancer, lung cancer, breast cancer, stomach cancer, colorectal cancer [73]
CA 72 − 4	≤ 6.90 KU/L	Serum, ascites	Stomach cancer, colorectal cancer, pancreatic cancer, breast cancer [74]
CA 24 − 2	≤ 15.00 U/mL	Serum	Liver cancer, stomach cancer, colon cancer, non-small cell lung cancer [75]
CA 15 − 3	≤ 24.00 KU/L	Serum	Breast cancer, lung cancer [76]
CYFRA 21 − 1	≤ 3.30 ug/L	Serum, pleural effusion	Lung cancer [77]
NSE	≤ 16.30 ug/L	Serum, spinal fluid, pleural effusion	Small cell lung cancer, neuroblastoma [78]
SCC	≤ 1.50 ng/mL	Serum	Squamous cell carcinoma of the lung, squamous epithelial cell carcinoma of the neck [79]

### MicroRNAs (miRNAs)

MicroRNAs (miRNAs) are short RNA sequences of approximately 20–24 nucleotides originating from non-coding genes, with over 28,645 variants identified to date [90, 91]. These molecules act as key regulators of gene expression, significantly impacting cancer pathogenesis by influencing cell proliferation, immune evasion, metastasis, and apoptosis [92–94]. Specific miRNAs, including miR-21, miR-141, miR-155, miR-26a, miR-27a, and miR-122, are well-documented in relation to oncogenic pathways [95, 96]. Emerging miRNAs, including miR-200c, miR-365, and miR-34a, show promise as early detection biomarkers due to their roles in inhibiting tumor cell migration, regulating epithelial-to-mesenchymal transition, and modulating immune surveillance. These functions highlight the clinical relevance of miRNAs as both biomarkers and active agents in cancer progression and response to treatment.

A distinctive feature enhancing miRNAs’ biomarker utility is their remarkable stability under physiological conditions; unlike conventional RNA markers, they resist enzymatic degradation and withstand temperature and pH fluctuations [97–101]. This stability allows miRNAs to persist in circulation and be detectable in bodily fluids such as plasma, serum, and saliva, promoting their use in non-invasive cancer diagnostics. To leverage these features, advanced microfluidic biosensors employing nucleic acid hybridization techniques have been developed for miRNA detection. These biosensors use DNA or RNA capture probes designed for specific miRNAs, facilitating highly specific hybridization within microfluidic channels. Microfluidic designs enhance sensitivity and detection efficiency, allowing rapid reaction process and minimizing reagent use. Detection methods following hybridization often involve fluorescence or electrochemical signals. For example, fluorescent labels on hybridized complexes allow quantification via emitted light, while electrochemical readouts convert miRNA binding into measurable electrical signals.

Most recent innovations regarding the use of biosensors to detect miRNAs in body fluids have been focused on integrating nanomaterials, such as gold nanoparticles and quantum dots, to amplify detection signals, thus enhancing assay sensitivity for detecting trace miRNA levels in patient samples. The integration of these technologies in microfluidic biosensors makes miRNAs powerful indicators in early cancer diagnostics, offering pathways for continuous disease monitoring, progression tracking, and assessment of treatment efficacy [10, 92, 94].

### CTCs

CTCs, representing individual cancer cells flowing through the bloodstream of cancer patients, have the potential to metastasize and establish secondary tumors in human body [102]. CTCs are a groupd of microscale cells, usually between 12 and 25 microns in diameters. While most CTCs succumb to apoptosis or phagocytosis within the bloodstream, a selective few manage to evade immune surveillance, adhere to other tissues, and proliferate extensively. This phenomenon results in the formation of metastatic foci, significantly increasing the mortality risk in cancer patients [103]. Consequently, CTCs are commonly regarded as the “seeds” of malignant tumor metastasis, making them pivotal biomarkers in cancer diagnosis.

Currently, the primary method of CTCs detection in blood involves immunological methods and immunofluorescence to isolate CTCs from blood samples, followed by morphological observation [104, 105]. However, the effectiveness of this approach is hampered by difficulties in the detection of CTCs caused by early-stage cancers. The limited number of CTCs in the bloodstream, together with the intricate isolation process and low detection sensitivity, renders the early detection of CTCs a formidable challenge. This inherent shortcoming significantly limits the potential of CTCs in early cancer screening. Addressing these challenges thus becomes imperative for exploiting the full diagnostic potential of CTCs and enhancing their effectiveness in early identification of cancer. Presently, the prevailing design paradigm for CTCs microfluidic devices is focused on leveraging their specificity to isolate CTCs from body fluids, followed by targeted detection of the isolated cells.

### Cell-free DNA (cfDNA) and circulating tumor DNAs (ctDNA)

cfDNA consists of DNA fragments released to bloodstream from various sources, including normal cell apoptosis, necrosis, and the turnover of healthy cells. It serves as a valuable biomarker for assessing various physiological and pathological conditions. ctDNA specifically refers to DNA fragments released into the bloodstream by cancer cells. These ctDNA fragments often show distinct characteristics, such as mutations, altered methylation patterns, and other cancer-specific genetic alterations, making them useful for studying the genetic composition and heterogeneity of tumors. As a subset of cfDNA, ctDNA provides insight into tumor dynamics, treatment response, and potential resistance mechanisms, thereby playing a critical role in the landscape of liquid biopsy for cancer diagnosis and monitoring [106, 107].

Currently, there is growing evidence supporting the presence and clinical relevance of cfDNA and ctDNA across various cancer types. cfDNA fragments average about 150–200 base pairs in length, corresponding to the size of nucleosome-bound DNA [108]. These fragments are released into bloodstream through cellular processes such as apoptosis and necrosis. ctDNA constitutes a small but crucial part of the total cfDNA pool, especially in patients with advanced cancers. Common cancer-related ctDNA includes mutations in genes such as TP53, KRAS, EGFR, BRAF, and PIK3CA, which are frequently altered in various malignancies [109].

The primary advantage of using cfDNA and ctDNA in liquid biopsies is also their ability to provide a non-invasive method of cancer detection and effective surveillance. Specifically, ctDNA can reflect the genetic variations within tumors, enabling the identification of particular mutations, chromosomal rearrangements, and other genomic aberrations associated with cancer progression and treatment resistance [110]. This capability aids in early detection, real-time monitoring of treatment responses, and identification of minimal residual disease, offering a comprehensive approach to cancer management. However, ctDNA concentrations in blood are often low, particularly in early-stage cancers, necessitating highly sensitive and specific assays. Currently, technologies such as digital droplet PCR (ddPCR) and next-generation sequencing (NGS) have demonstrated potential in detecting low levels of ctDNA. In ddPCR, the sample is divided into tens of thousands or even millions of microdroplets, each serving as an independent PCR reaction site to amplify minute amount of target DNA. This setup enables the amplification of a single or few molecules per droplet, enhancing ddPCR’s sensitivity in detecting low-abundance ctDNA. After amplification, the absolute copy number of target DNA is determined by measuring fluorescence intensity in each droplet, making ddPCR suitable for detecting rare mutations or low ctDNA concentrations [111]. In contrast, NGS uses a high-throughput sequencing platform to simultaneously sequence a large number of DNA molecules. ctDNA samples are first fragmented, followed by the addition of adapters. After that, the fragments are amplified and sequenced on a solid surface. NGS has the advantage of comprehensively detecting a wide range of mutations, copy number variations and rearrangements, making it an effective tool for identifying diverse molecular markers in tumors [112]. Thus, ddPCR and NGS provide complementary approaches, with ddPCR ideal for absolute quantification and NGS for complex mutation profiling in ctDNA analysis. Despite this, further enhancements in sensitivity, specificity, and cost-effectiveness are required to make these technologies widely accessible [113]. Additionally, the biological complexity of ctDNA, including its fragmented patterns and interactions with other biomolecules in the blood, poses challenges that limit its widespread application.

Concerning ctDNA, current research is focused on enhancing the analytical performance of cfDNA and ctDNA detection methods. Microfluidic platforms can handle extremely small volumes of blood, which is particularly beneficial for rare biomarker detection such as ctDNA. This precise fluid control allows for efficient sample preparation, reducing the loss of ctDNA and improving the overall assay sensitivity. Additionally, microfluidic devices can integrate multiple processing steps, such as cell separation, DNA extraction, and amplification, into a single platform, thus streamlining the workflow and reducing the risk of contamination. The integration with ddPCR allows for the absolute quantification of ctDNA, providing precise measurements of ctDNA concentrations without the need for standard curves. This high sensitivity is crucial for detecting low levels of ctDNA in early-stage cancers. NGS, on the other hand, offers comprehensive profiling of ctDNA, enabling the detection of a broad range of genetic alterations, such as point mutations, insertions, deletions, and structural variations. The combination of microfluidics with NGS can thus provide a detailed and accurate characterization of the tumor genetic landscape from a minimal blood sample. The integration with ddPCR allows for the absolute quantification of ctDNA, providing precise measurement of ctDNA concentrations without the need for standard curves. This high sensitivity is crucial for detecting low levels of ctDNA in early-stage cancers. NGS, on the other hand, offers comprehensive profiling of ctDNA, enabling the detection of a broad range of genetic alterations, such as point mutations, insertions, deletions, and structural variations. The combination of microfluidics with NGS can thus provide a detailed and accurate characterization of the tumor genetic landscape from a minimal blood sample.

Recently, there are a few studies that have explored the integration of microfluidic technology with ddPCR and NGS for ctDNA detection. These studies aim to leverage the strengths of each technology to improve the sensitivity, specificity, and efficiency of ctDNA assays [114, 115]. However, the field is still not very mature. The primary reasons for the limited number of such studies include the technological challenges mentioned earlier, as well as the need for interdisciplinary collaboration between engineers, biologists, and clinicians. Developing these integrated platforms requires expertise in microfluidic design and fabrication, molecular biology, and clinical diagnostics. Additionally, there are regulatory and standardization challenges that need to be addressed to ensure the reliability and reproducibility of these integrated assays in clinical practice.

In summary, while the integration of microfluidic technology with ddPCR and NGS holds great promise for the sensitive and specific detection of ctDNA in blood, significant challenges remain. Continued research and development, along with interdisciplinary collaboration, are essential to overcome these challenges and fully realize the potential of these advanced diagnostic platforms.

### Other biomarkers

In addition to the aforementioned biomarkers, recent research has introduced several other common nano-markers with diagnostic potential for cancer, such as nucleases and volatile organic molecules [116, 117]. Among them, telomerase, a type of nuclease, has attracted attention. Abnormal enhancement of telomerase activity in certain cancer cells could result in the prolonged lifespan of these cells, thereby promoting cancer cell proliferation [118]. Typically functioning intracellularly, significant amounts of telomerase in cancer cells are released into body fluids including blood and urine, eliciting an immune response. This release can be detected using the Telomeric Repeat Amplification Protocol method, making telomerase a promising biomarker for early cancer diagnosis and prognosis [119]. Moreover, organic molecules present in body fluids may originate from cellular metabolism, manifesting as volatile organic compounds (VOCs) generated due to oxidative stress or membrane peroxidation resulting from genetic or protein mutations in cancer cells [120]. These organic molecules can also reflect metabolic changes induced by inflammation, cell necrosis, and the growth or degeneration of tumor tissue. For instance, certain polyamines, alkanes, and olefins have been identified at elevated levels in the blood of cancer patients compared to non-cancer counterparts. The structural simplicity and stability of these compounds facilitate their identification, presenting advantages over more complex biomolecules as potential markers [121]. However, the current limitation lies in the relatively low specificity of these markers for cancer identification, preventing their application in early cancer detection at present. Continued research efforts aim to refine the specificity of these markers, unleashing their potential for enhanced precision in early cancer diagnosis.

It is important to emphasize that although numerous molecular biomarkers are available for cancer detection, simply relying on a single biomarker measurement lacks the specificity needed for accurate cancer diagnosis. This limitation arises from the non-disease-specific nature of many biomarkers, which may show similar upregulation or downregulation patterns in non-cancerous diseases or across different cancer subtypes. For example, CEA, whose amount is frequently elevated in colorectal, pancreatic, and breast cancers, could also increase in inflammatory bowel disease, chronic obstructive pulmonary disease, and liver cirrhosis due to inflammation and tissue damage. Similarly, PSA, a critical marker for prostate cancer, may increase in benign prostatic hyperplasia and prostatitis due to non-malignant cell proliferation and inflammation. AFP, used in liver cancer diagnostics, can be elevated in chronic hepatitis, liver cirrhosis, and even pregnancy, as liver cell regeneration in non-cancerous liver diseases also elevates AFP levels. Furthermore, ctDNA can provide valuable insight into cancer presence and progression, yet they are not exclusively cancer-specific. For instance, cfDNA, a broader category that includes ctDNA, can be released in response to cell death or inflammation in non-cancerous diseases such as autoimmune conditions, hepatitis, and sepsis, complicating cancer diagnosis. Similarly, miRNAs, though valuable in cancer diagnosis, are also non-specific. For example, miR-21 is elevated in various cancers, including breast, lung, and colorectal cancer, yet it also increases in cardiovascular disease and rheumatoid arthritis due to its role in inflammation. miR-155, upregulated in cancers such as lymphoma and breast cancer, is also elevated in autoimmune diseases like lupus and multiple sclerosis due to immune modulation functions. miR-122, typically a liver cancer marker, rises in hepatitis and liver cirrhosis as a response to liver cell injury [67]. Consequently, cancer-associated biomarkers may also be elevated in non-cancerous conditions, introducing diagnostic ambiguity. While microfluidic platforms are powerful tools for biomarker analysis, accurate cancer diagnosis frequently requires the combined analysis of multiple biomarkers approach to improve diagnostic specificity and precision.

Due to the advantages of combined detection, researchers have developed several biosensors capable of simultaneously detecting multiple biomarkers. These biosensors can be broadly categorized into two types: those that detect multiple biomarkers of the same type and those that detect different types. Detecting multiple biomarkers of the same type enables the simultaneous detection of multiple serum protein markers, facilitating a more accurate assessment of tumor load, metabolic activity, and the immune microenvironment in cancer patients. This centralized analysis permits simultaneous observation of expression changes in different markers within similar biological pathways, revealing subtle biological differences and enhancing diagnostic accuracy and sensitivity to cancer progression. Moreover, during dynamic monitoring, this centralized assay tracks cellular and molecular responses to cancer treatment in real time, allowing for rapid assessment of fluctuations in specific proteins or peptides to evaluate treatment efficacy. For instance, the ExoSearch chip developed by Zhao et al. significantly enhances ovarian cancer diagnosis by labeling three exosomal tumor markers (CA-125, EpCAM, and CD24) with fluorescent dyes and performing simultaneous measurements of these markers in a single channel [122].

The second type includes biosensors capable of simultaneously detecting different biomolecules (e.g., proteins, nucleic acids, and cells). By integrating various biomarkers, including proteins, DNA, and RNA, these biosensors comprehensively reveal the biological characteristics of cancer across different developmental stages and provide valuable insight at various histological levels. This comprehensive analysis is crucial for early cancer diagnosis, as different biomarker types may change at different time points during cancer development, making accurate disease state assessment through single-marker testing unfeasible. Furthermore, combining multiple markers can underpin individualized therapy by analyzing the mutation and epigenetic modification status of tumor cells during treatment to inform targeted therapeutic strategies. In this context, Zhou et al. developed a dual-channel integrated 3D microfluidic device for simultaneous multiplexed and sensitive in situ detection of two types of exosomal biomarkers (proteins and miRNAs). This biosensor accurately distinguishes between cancerous and normal cells while successfully differentiating cancer cell subtypes and monitoring cancer staging [123].

Despite the significant advantages offered by both types of biosensors in cancer detection, they encounter several challenges. The technological complexity not only complicates device integration and data analysis but also entails high costs and substantial resource requirements. Additionally, precise data integration algorithms are essential to ensure diagnostic accuracy and mitigate cross-reactivity. Future research should prioritize optimizing sensor design and material selection to improve detection specificity and reduce cross-reactivity. Advancements in nanotechnology and microfabrication processes are anticipated to significantly reduce the cost and resource consumption of multi-detection platforms, enhancing their economic viability.

## Microfluidic biosensors for early cancer detection

After establishing a thorough understanding of the fundamental principles, fabrication techniques, materials, and various tumor markers employed in microfluidic devices, we will present detailed examples of cancer detection applications using different microfluidic biosensors to improve readers’ understanding of the practical application of these concepts.

Currently, there are a variety of biosensors being applied to cancer detection. Among these, electrochemical biosensors, surface-enhanced Raman spectroscopy (SERS) biosensors, and immunosensors have been most extensively studied. The primary function of these biosensors is to utilize their high sensitivity and specific recognition capabilities for particular biomarkers in order to achieve rapid and accurate detection of cancer-related biomarkers. They convert the recognition events of specific biomolecules in pre-treated samples into measurable signals through various signal transduction mechanisms such as electrochemical, optical, mechanical methods etc., enabling early diagnosis and monitoring of cancer. These analytical tools exhibit diversity based on different detection principles (e.g., surface plasmon resonance, electrochemistry, and immunosorbent), utilizing different signal amplification strategies and adapting to various sample types and assay environments. This provides a wide range of selectivity and flexibility for cancer detection. In general, microfluidic devices used for early cancer detection need to be integrated with the biosensor technologies described above to accomplish separation and detection in an integrated manner.

**Fig. 2:**
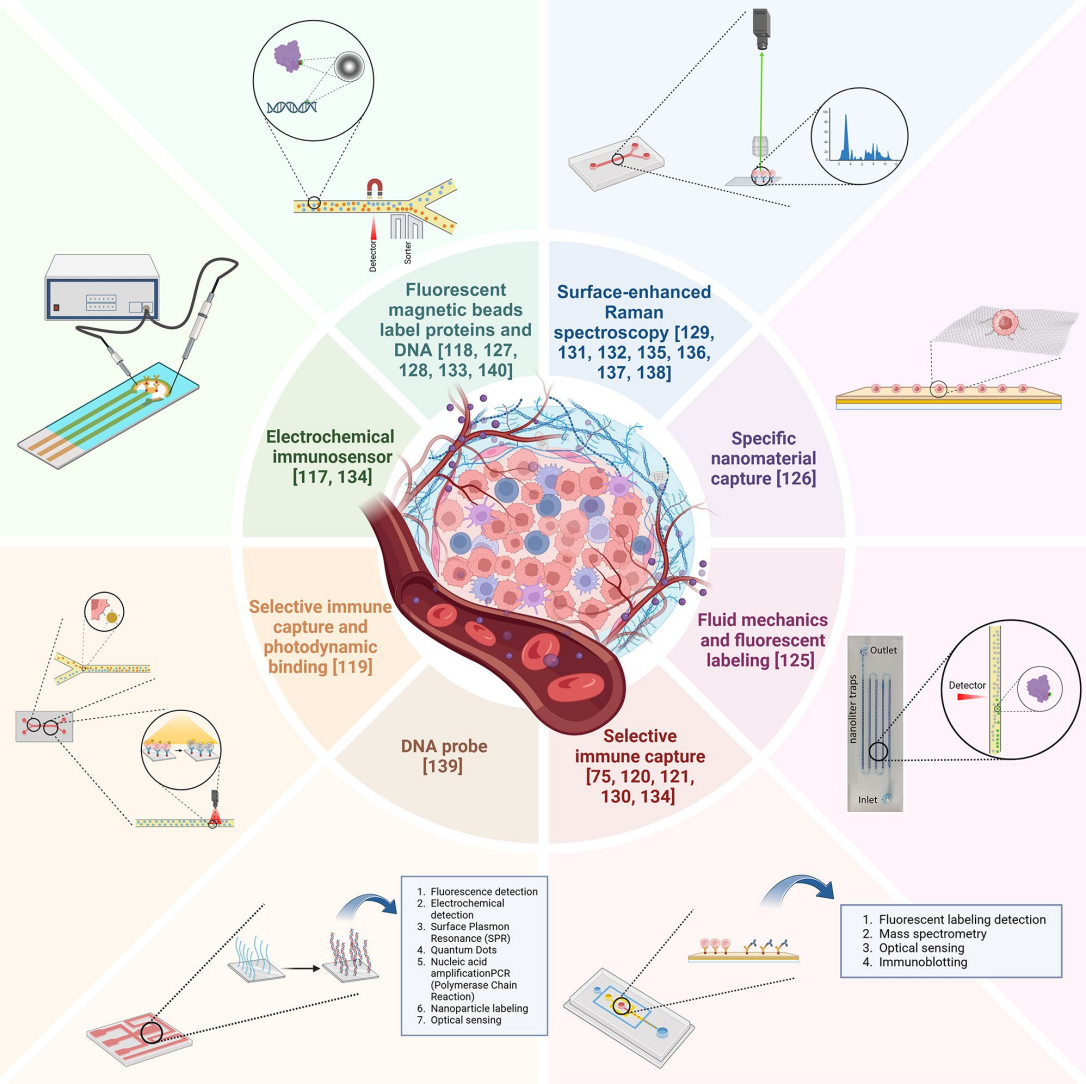
Characteristics of microfluidic devices developed in the last five years for early cancer detection

The evolution of science and technology has resulted in a deeper understanding of cancer, with the continuous discovery of new cancer types and pathogenic mechanisms. Simultaneously, the progression of microfluidic device fabrication technology and the persistent exploration of diverse tumor nano-markers have spurred the creation of many microfluidic devices tailored for early cancer detection. This part encompasses the representative microfluidic devices for nano-biomarker detection developed in the last five years [87, 124–145]. These have been systematically categorized based on different detection techniques (Fig. 2), and a few of the main types are described in detail.

### Microfluidic biosensors combining microfluidics and electrochemical sensors

Microfluidic electrochemical sensing systems typically comprise miniaturized fluidic channels, electrodes, and electrochemical detection interfaces. Fluids traverse the chip by capillary effect or other actuation mechanisms such as pressure-driven flows or electrophoresis to facilitate sample transport, mixing, and reaction. Electrochemical sensors measure signals from redox reactions, translating molecular recognition into measurable changes in electric current, voltage, or resistance. Electrochemical sensors combined with microfluidic devices include immunosensors, aptamers (a collective term for a molecule that specifically binds to a target molecule in a sensor system), and capacitive sensors. These sensors can be embedded directly into the microfluidic device. What’s more, multiple electrodes can be arranged within the microfluidic channel to form an electrode array to create a complete detection device. The introduction of microfluidic systems has markedly improved the sensing parameters of these devices, such as detection limit, response time, and specificity, by ensuring precise control of fluid dynamics and enhancing the interaction between the sensor and the analyte.

**Fig. 3:**
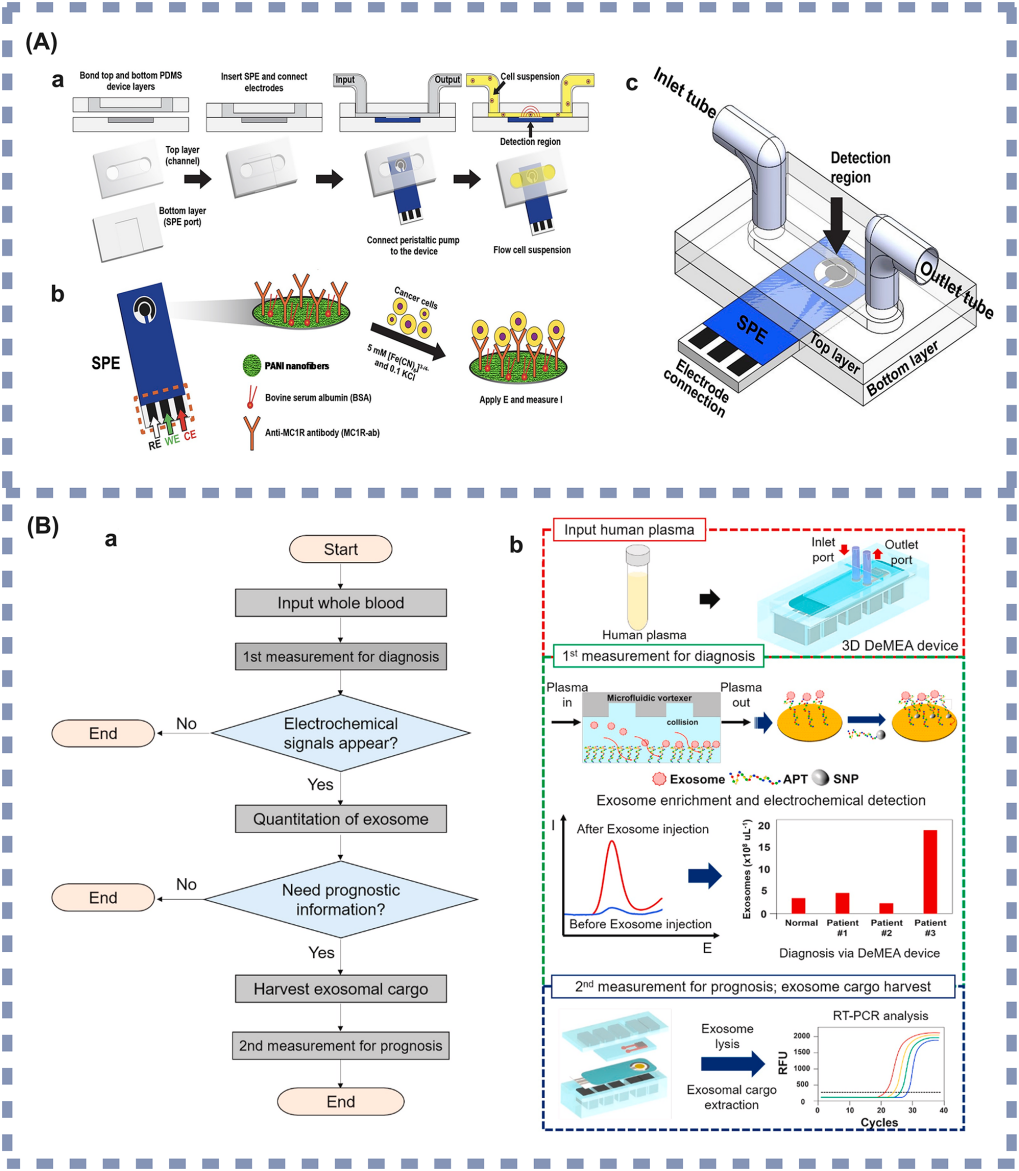
(**A**) Microfluidic devices based on electrochemical immunosensors (**a**) Microfluidic device components and assembly sequence. (**b**) Scheme for MC1R-Ab-PANI/SPE electrochemical immunoassay for melanoma cell detection. (**c**) The assembled microfluidic device. Reprinted with permission [124]. Copyright (2019), with permission from Elsevier. (**B**) Microfluidic devices based on electrochemical aptamer sensors (**a**) The workflow chart for sequential analysis of cancerous sEVs. (**b**) Schematics of complete analysis system. Reprinted with permission [139]. Copyright (2020), with permission from Elsevier. MC1R: Melanocortin 1 receptor. Ab: Antibodies. PANI: Polyaniline nanofiber. SPE: Screen-printed electrode. DeMEA: Electrochemical apta-sensor. APT: Aptamer. SNP: Silver nanoparticles

In recent advancements, researchers have mainly focused on electrochemical immunosensors and aptamer sensors. Electrochemical immunosensors utilize electrochemical signals induced by the interaction of antibodies or antigens with target molecules and reflect the concentration of the target molecule to be measured by recognizing changes in this electrical signal. Microfluidic biosensors formed by combining such sensors with microfluidic devices have been reported. For example, Prathap et al. [124] integrated an electrochemical immunosensor within a microfluidic system to detect early melanoma. Their device incorporated channel filters of specific pore sizes that selectively allowed CTCs to pass while filtering out larger cells. For post-separation, the device employed Melanocortin 1 receptor (MC1R) as a biomarker for cancerous cells, using antibodies that could covalently bind to a polyaniline (PANI) nanofiber-modified screen-printed electrode (SPE). Exposure of this antibody-modified electrode to a sample containing melanoma cells resulted in the formation of antibody-antigen complexes, enabling melanoma cell detection through measured changes in the electrochemical signal (shown in Fig. 3(A)). The biosensor uses “buffer mixed with tumor cells” and “peripheral blood mononuclear cells (PBMCs) mixed with tumor cells” as test samples, in which a low detection limit of 10 cells/10 mL is achieved. Given its sample type and low detection limit, this microfluidic biosensor is suited for various clinical applications, including early melanoma screening, monitoring of high-risk populations, assessment of patient recurrence, individualized treatment adjustments, and clinical research. These applications can improve the detection and monitoring of melanoma, and improve our understanding of the mechanisms of immune response in the tumor microenvironment, providing valuable insight for the development of new therapeutic strategies.

Aptamer sensors utilize an aptamer to bind to a target molecule with high specificity. Based on this, Kashefi-Kheyrabadi and coworkers developed a detachable electrochemical aptamer sensor. They combined it with microfluidics to form a microfluidic aptamer biosensor for analyzing breast cancer cellular epithelial cell adhesion molecule (EpCAM) in plasma [139]. The device utilized nanostructured electrochemical aptamers and microfluidic technology for highly sensitive, specific, and rapid detection of small sEVs. A material binding specifically to EpCAM served as an electrochemical aptamer, immobilized on the sensing surface of the microfluidic device to capture exocytosis proteins and form complexes. The unique design of the microfluidic channel increased the collision probability between the sEVs and the aptamer on the sensing surface, thus facilitating their binding. sEVs captured on the sensing surface were detected by introducing a secondary EpCAM aptamer labeled with silver nanoparticles (AgNPs). The concentration of sEVs was then determined by monitoring the oxidation of the silver nanoparticles, generating an electrochemical signal proportional to the number of sEVs captured on the sensing surface (shown in Fig. 3(B)). This microfluidic biosensor is optimized for early breast cancer screening, postoperative monitoring, and routine testing of high-risk individuals. The biosensor utilizes plasma as the sample medium, with a detection limit as low as 17 sEVs/µL, enabling sensitive detection of even trace amounts of breast cancer-associated sEVs in circulation. As such, the sensor is suitable for non-invasive liquid biopsy applications, facilitating real-time monitoring of tumor progression or recurrence. It is especially valuable for postoperative or previously treated patients, providing rapid detection of potential relapse. Additionally, individuals with a familial history of breast cancer or other high-risk factors may undergo regular screenings using this sensor, thereby enhancing early diagnostic accuracy and minimizing the need for invasive tissue biopsies.

In practical applications, both immunosensors and aptamer sensors exhibit high specificity, ensuring accurate recognition of target molecules. Theoretically, both sensors have high sensitivity, allowing the detection of low concentrations of target molecules. Additionally, they both offer certain degree of customizability. The design and selection of antibodies and aptamers can be tailored according to specific applications, enhancing detection specificity and efficiency [146]. The clinical potential of this type of microfluidic biosensors therefore lies in their ability to rapidly and accurately quantify specific cancer-associated molecules such as nucleic acids and proteins from the smallest sample sizes. This makes them suitable for immediate diagnosis and continuous monitoring in future clinical setting.

However, differences also exist between these two types of sensors, potentially rendering them suitable for different requirements. These distinctions encompass the production process: the generation of antibodies used in immunosensors often involves intricate biological procedures such as animal immunization, cloning, and purification, making it potentially more costly. In contrast, aptamers are typically synthesized chemically, employing a comparatively simpler and potentially more cost-effective production process. Regarding biological source, antibodies used in immunosensors are typically obtained from animal immunization, thus raising concerns related to animal use and ethical considerations. Conversely, aptamers are generated in vitro through in vitro screening or synthesis, circumventing the need for animal use and aligning with ethical and regulatory standards. Concerning commercial availability, a broad spectrum of antibodies is industrially accessible, albeit with potential cost implications for specific customizations. Aptamers, on the other hand, usually require in vitro screening or laboratory design, resulting in relatively lower commercial availability, potentially requiring additional research efforts. In terms of application scope, immunosensors primarily target biological molecules such as proteins and antigens. Aptamer sensors, on the other hand, are more versatile in that they are suitable for a broad array of biomolecule types, including proteins and nucleic acids, thus expanding their range of applications. Assessing maturity, antibodies as biomolecules have undergone extensive study and application, resulting in the availability of numerous commercial products. In contrast, aptamer technology is evolving, with commercial availability potentially lagging behind and being relatively limited [119, 147].

In summary, the combination of microfluidic devices and electrochemical sensors holds significant promise in early cancer detection by improving the early diagnosis rate of cancer and providing more accurate biomarker information for personalized treatment. However, there are some common problems with this type of device, such as the susceptibility of the microelectrode surface to interference by nonspecific adsorption, variations in electrochemical signals due to changes in electrolyte, pH fluctuations, or the presence of organic solvents. Signal enhancement strategies may also contribute to system complexity [148]. Therefore, the research and development of (1) new types of microfluidic devices, (2) electrochemical sensors that incorporate anti-pollution materials, (3) effective electrode cleaning methods, and (4) optimized detection systems are necessary. This includes increased monitoring and regulation of environmental parameters to reduce their influence on cancer detection.

### Microfluidic biosensors combining microfluidics and surface-enhanced raman spectroscopy (SERS)

SERS is a technique that utilizes surface nanoparticles to significantly amplify Raman signals. By introducing metal nanoparticles near the molecule to be detected through the near-field effect, the localized surface plasmon resonance (LSPR) of these metal nanoparticles excites an electromagnetic field, known as a “hot spot”, leading to a substantial enhancement in the Raman scattering signal. The ‘hot spot’ typically emerges in the gap between closely spaced nanoparticles, necessitating the careful selection of suitable metal nanoparticles and the precise fabrication of a microfluidic chip [149]. The microfluidic device precisely controls the flow of droplets to direct the sample to the region containing the metal nanoparticles and retain them in the region for a suitable time period, ensuring the acquisition of enhanced Raman spectra. SERS technology enables ultra-high sensitivity detection of tumor-associated molecules, including free DNA fragments in blood, sEVs, and specific tumor signature proteins. The introduction of microfluidic systems in SERS applications further refines the sensitivity and selectivity of the detection process, enhancing the interaction between the target analyte and the ‘hot spots’ for more accurate readings.

**Fig. 4:**
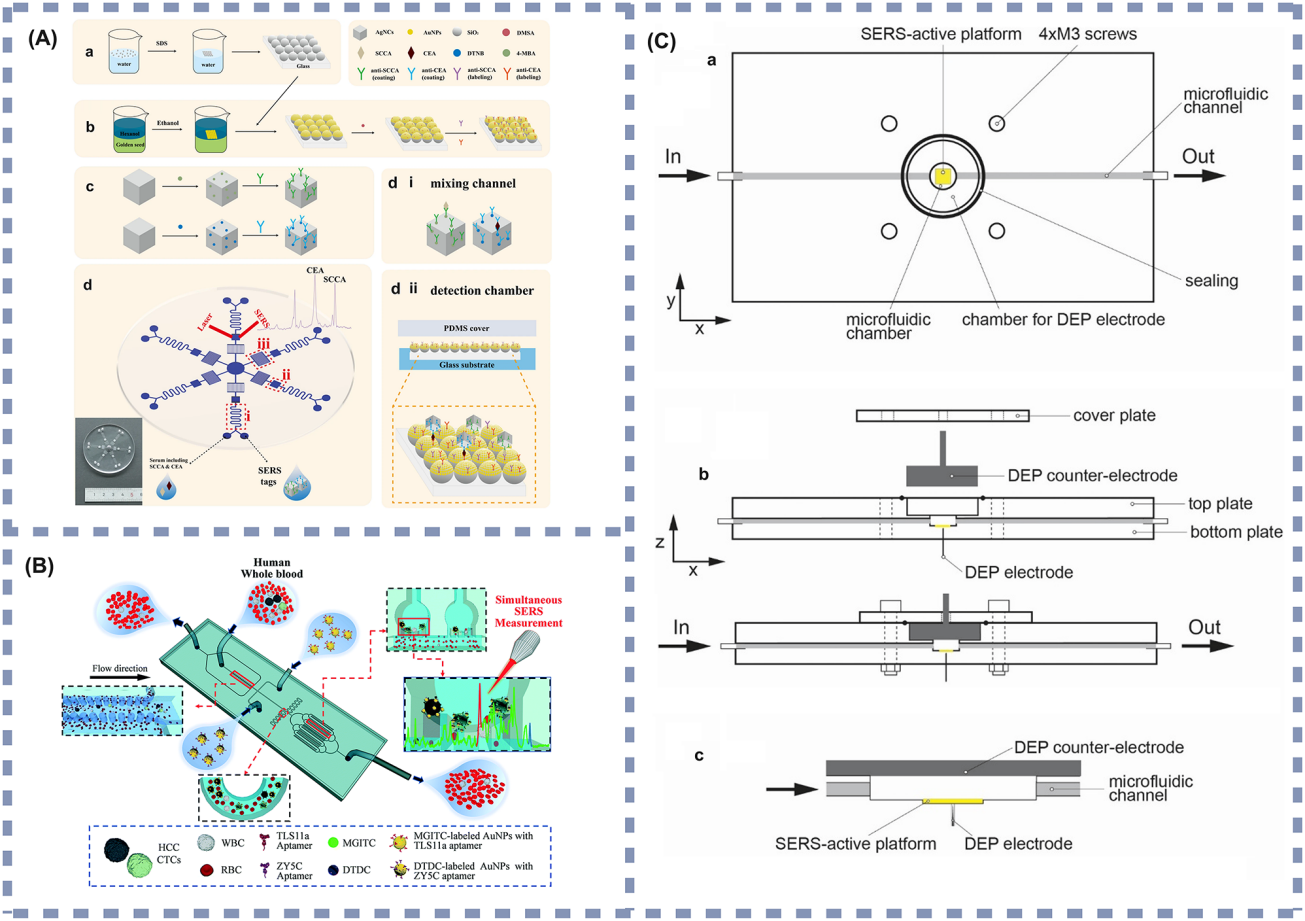
(**A**) Microfluidic device using Au nanoparticle–modified SiO_2_ microsphere (Au@SiO_2_) array–based highly active SERS substrates. **a**: Preparation of SiO_2_ array. **b**: Preparation of Au@SiO_2_ array substrate and capturing substrate. **c**: Fabrication of SERS tags. **d**: Schematic illustration of the pump-free microfluidic chip for SERS detection of SCCA and CEA. The chip contained three functional sections: (i) mixing channel, (ii) detection chamber. Reprinted with permission [134]. Copyright (2022), with permission from Springer; (**B**) The workflow of SERS-aptamer based microfluidic chip for hepatocellular carcinoma CTCs capture, SERS measurement, and single-cell phenotype analysis. Reprinted with permission [136]. Copyright (2021), with permission from Royal Society of Chemistry; (**C**) Schematic view of the microfluidic chip for the dielectrophoretic deposition of CTCs on the surface of the SERS platform (**a**), cross-section of the chip before assembly and after placing cover plate on the top plate (**b**), detailed view of the microfluidic chamber with the DEP electrode under the SERS platform and counter-electrode at the top of the chamber (**c**). Reprinted with permission [137]. Copyright (2022), with permission from MDPI. SERs: Surface-enhanced Raman scattering. SCCA: Squamous cell carcinoma antigen. CEA: Carcinoembryonic antigen. SDS: Sodium dodecyl sulfate. AgNCs: Ag nanocubes. AuNPs: Au nanoparticles. DMSA: Dimercaptosuccinic acid. DTNB: 5,5’-dithiobis-2-nitrobenzoic acid. 4-MBA: 4-mercaptobenzoic acid. PDMS: Polydimethylsiloxane. HCC: Hepatocellular carcinoma. CTCs: Circulating tumor cells. WBC: White blood cells. RBC: Red blood cells. MGITC: Malachite green isothiocyanate. DTDC: 3,3′-diethylthiadicarbocyanine iodides. DEP: Dielectrophoresis

In recent years, researchers have developed various SERS-based microfluidic biosensors that differ in the types of metal nanoparticles used for SERS, and in the methods of combining SERS with microfluidic devices. Commonly used nanoparticles include silver, gold, and copper nanoparticles. Regarding the binding method, in recent years the most common approach integrates the microfluidic channel and SERS-active substrate directly on the same chip. Based on this approach, researchers fabricated a microfluidic biosensor featuring a highly SERS-responsive Au@SiO_2_ array substrate coupled with Ag nanocubes (AgNCs) [134]. This platform immobilized serum biomarkers onto the Au@SiO_2_ arrays through immunoassay techniques. The orderly nano-gaps within the Au@SiO_2_ arrays produced numerous “hot spots” with AgNCs, substantially boosting the SERS signals (shown in Fig. 4(A)). The detection limits for squamous cell carcinoma antigen (SCCA) and CEA in human serum reached 0.37 pg/mL and 0.28 pg/mL, respectively. This microfluidic biosensor utilizes serum as the sample medium and exhibits exceptionally low detection limits, allowing for the highly sensitive detection of trace amounts of SCCA and CEA. As such, the sensor is suited for non-invasive liquid biopsy applications and is appropriate for early diagnosis, postoperative monitoring, and screening of patients with squamous cell carcinoma, as well as other cancers such as colorectal and lung cancer, especially in high-risk populations. This technology enables physicians to monitor tumor progression or recurrence in real time. For individuals in high-risk groups or those with a familial predisposition, regular screening with this sensor can enhance early diagnostic efficiency and help prevent delays in disease detection.

Similarly, Szymborski et al. [137]. developed a SERS microfluidic device based on the dielectrophoretic effect for the specific detection of breast CTCs in the plasma of breast cancer patients. The device, relying on a SERS-active substrate modified with silicon and AgNCs, specifically captured cancer cells on a SERS chip through the dielectrophoretic effect, achieving a detection limit of 20 cells/mL (shown in Fig. 4(C)). This microfluidic biosensor demonstrates high sensitivity to low concentrations of plasma CTCs, making it suitable for early screening and recurrence monitoring in high-risk populations, including individuals with a familial history of breast cancer, known genetic mutations (e.g., BRCA1/2), or those already diagnosed and requiring treatment efficacy monitoring. As plasma is the test sample, the collection process is minimally invasive and requires only simple procedures, making the biosensor suited for routine medical checkups, outpatient follow-ups, and frequent monitoring scenarios. With a detection limit of 20 cells/mL, the biosensor enables the efficient capture of low concentrations of circulating tumor cells, making it useful for early detection of developing cancer or for continuous monitoring during postoperative and chemotherapy periods to assess disease progression and treatment response.

In addition, there are microfluidic biosensors that modify the microfluidic channels with nanostructures. In this approach, appropriate metallic nanostructures are introduced on the surface of the microfluidic channel, allowing the sample flowing through the channel to interact with these structures and resulting in an enhanced Raman signal in the SERS detection region. For example, Gao et al. [136] utilized AuNPs as substrate nanomaterials and prepared multivalent SERS-streptavidin nanotags, developing a SERS microfluidic device with a lantern-like bypass microchannel structure for the quantitative analysis of hepatocellular carcinoma (HCC) CTCs in whole blood samples. The device efficiently captured CTCs from whole blood samples by size separation through its unique lantern-like bypass structure, followed by SERS-streptavidin nanostructures that specifically bound to CTCs and allowed their immobilization on AuNPs substrates (shown in Fig. 4(B)). The average capture rate of the device was 84%. This microfluidic biosensor is optimized for early screening and ongoing disease monitoring in high-risk individuals and patients already diagnosed with hepatocellular carcinoma (HCC). High-risk populations include patients with chronic hepatitis B or C, individuals with chronic alcohol use, and those with cirrhosis. Whole blood is used as the sample, which is easily obtained and requires minimal processing, thereby enhancing clinical workflow efficiency. Additionally, the sensor exhibits high sensitivity in detecting circulating tumor cells (CTCs). As such, the device is suitable for routine checkups, early cancer screening, and evaluating treatment efficacy. It facilitates real-time monitoring of tumor burden and metastasis risk, providing critical information for personalized treatment strategies.

Both methods mentioned above have the advantages of high integration and high sensitivity in practical applications. However, notable differences exist in their application complexities. For instance, in terms of preparation complexity, the direct integration of microfluidic channels and SERS active substrates on the same chip may necessitate a more precise preparation process, thus imposing greater demands on the overall preparation procedure. Regarding nanostructure modification, it is relatively easy to achieve the preparation of microfluidic channels. The applicability of these methods varies. Directly integrated structures are better suited for scenarios requiring precise control of the microfluidic channel structure and simultaneous SERS detection on the same chip. On the other hand, nanostructure-modified microfluidic channels offer greater flexibility, making them easier to apply to different sizes and shapes. When considering reusability and stability, structures directly integrated on the same chip tend to exhibit better stability and reusability for long-term experiments and multiple uses. However, nanostructure modifications require careful attention to stability and may necessitate periodic adjustments to maintain sensor performance in certain cases. In terms of cost, directly integrated structures may incur higher preparation costs but may demonstrate superior performance and reliability in high-end applications. Conversely, nanostructure modifications are relatively easier to implement and are a more cost-effective option for applications with budget constraints [150–152].

In summary, the SERS-based microfluidic device demonstrates the capability for both single- and multi-molecule detection of cancer markers with high sensitivity. Importantly, it eliminates the need for complex sample pretreatment, contributing to early-stage cancer diagnosis. The clinical significance of this type of microfluidic biosensors lies in their ability to provide detailed molecular information that enhances the identification and characterization of cancer cells. SERS-based microfluidic devices are particularly valuable for early cancer detection and for differentiating between different types of cancer, allowing for more personalized treatment plans. Nevertheless, the SERS signal is influenced by various factors, including the shape, size, and arrangement of metal nanoparticles, and the preparation and operation techniques of the reagents demand specialized spectrometer equipment with higher costs. Despite these challenges, the combination of SERS and microfluidics represents a significant advancement in the field of biosensing, providing a robust platform for the sensitive and specific detection of cancer biomarkers.

### Microfluidic biosensors combining microfluidics and magnetic-based enrichment

**Fig. 5:**
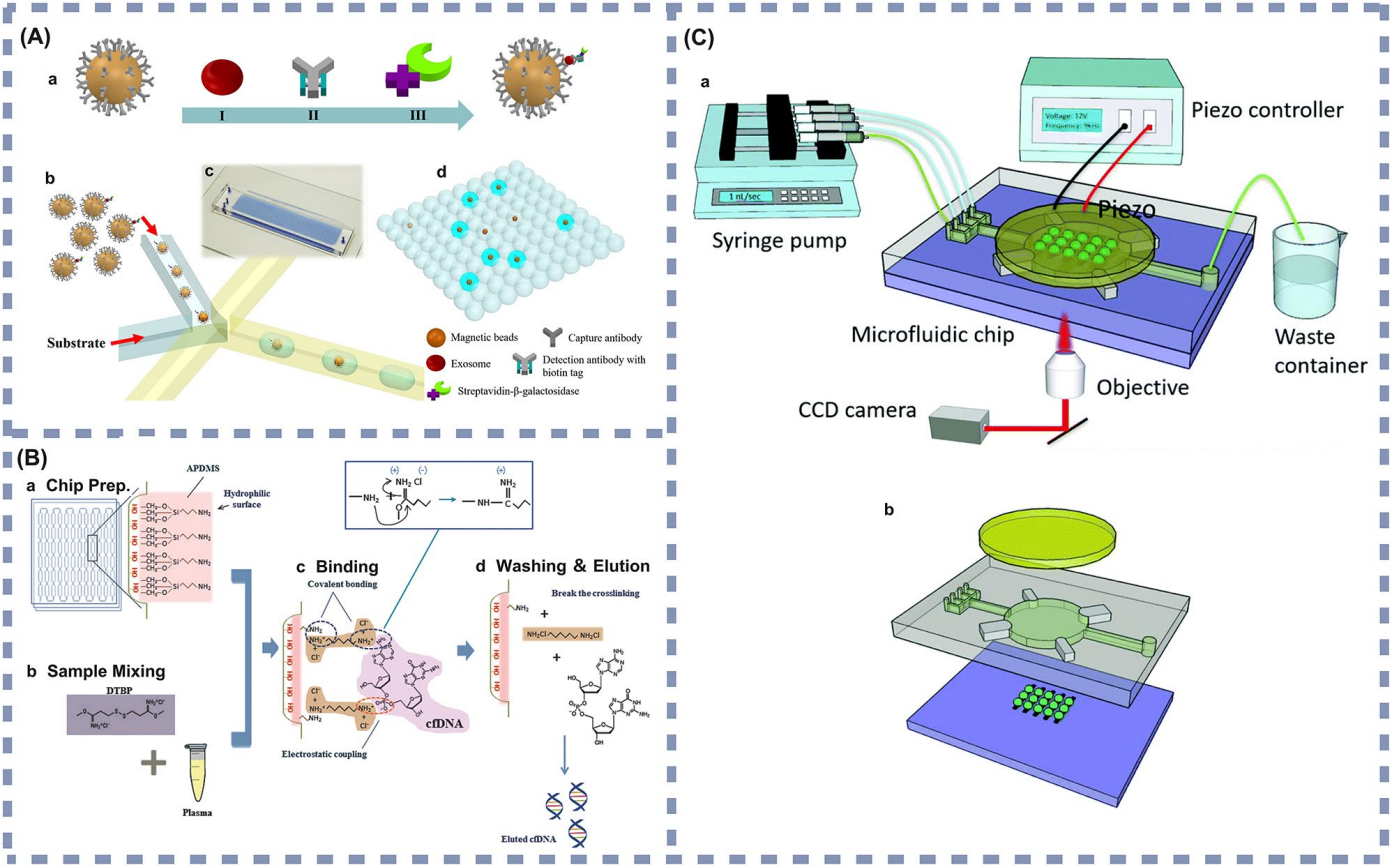
(**A**) Schematic showing the droplet digital ExoELISA for sEVs quantification. **a**: Single sEVs immunocomplex constructed on a magnetic bead. **b**: Substrate and beads are co-encapsulated into microdroplets. **c**: Droplet digital ExoELISA chip. d: Fluorescent readout for counting the positive droplets with the target sEVs. Reprinted with permission [132]. Copyright (2018), with permission from American Chemical Society; (**B**) Operation principles of a cfNAs isolation based microfluidic system with DTBP. **a**-**d**: Chip preparation and detection steps. Reprinted with permission [133]. Copyright (2018), with permission from Wiley; (**C**) A multiplexed microfluidic biomarker detection system. **a**: A schematic drawing of the detection system. **b**: The microchip structure with a PDMS layer and a glass layer. **c**: The micrograph of the detection chamber with four air bubbles. Reprinted with permission [145]. Copyright (2018), with permission from Royal Society of Chemistry. ExoELISA: Single- sEVs-counting enzyme-linked immunoassay. cfNAs: Cell-free nucleic acids. DTBP: Dimethyl Dithiobispropionimidate. APDMS: Aminopropyl polydimethylsiloxane. cfDNA: Cell-free DNA. PDMS: Polydimethylsiloxane. CCD: Charge coupled device

Magnetic-based enrichment strategies, including the use of immunomagnetic beads and other magnetic particles, have been demonstrated to be effective in the isolation and concentration of target biomarkers within microfluidic systems. These magnetic particles are engineered to specifically interact with biomolecules such as proteins, nucleic acids, and other cancer-related markers by functionalizing their surfaces with antibodies, aptamers, or ligands that selectively bind to the intended targets [153]. In microfluidic devices, magnetic enrichment involves introducing these functionalized magnetic particles into the microchannel, where their movement is controlled by an external magnetic field to capture and isolate the desired biomarkers. This approach enables the precise and efficient concentration of low-abundance molecules from complex biological samples. Once captured, the enriched biomarkers are subsequently transported to the detection region within the microfluidic device via the microchannels. Furthermore, magnetic-based enrichment techniques can readily be coupled with various detection modalities, significantly enhancing signal sensitivity. For example, secondary labeling methods including fluorescent, enzymatic, or electrochemical signals can be employed to amplify the detection signal. These integrated systems provide high specificity and sensitivity, facilitating cost-effective, automated, and high-throughput analysis. For example, a magnetic bead pellet coupled with a CD63 antibody was utilized to capture target sEVs (Glypican-1 (GPC-1)) from the plasma of early-stage breast cancer patients [132]. The development of a microfluidic biosensor using this magnetic bead utilizes droplet microfluidics to achieve highly accurate detection of target sEVs. The magnetic beads are modified to carry a CD63 antibody capable of specifically recognizing proteins on the surface of sEVs. These magnetic beads are mixed with sEVs and separated magnetically to ensure that each target sEVs can be captured by a single magnetic bead. After formation of individual sEVs-magnetic bead complexes, they are further recognized and formed into enzyme-linked immunocomplexes with them using detection antibodies with enzymatic markers. Next, these complexes are introduced into a microfluidic biosensor chip. In the sensor, the magnetic beads and the enzyme substrate are co-encapsulated into tiny droplets with a diameter of 40 micrometers, precisely controlled by the Poisson distribution principle. These droplets are dispersed in mineral oil, which catalyzes the generation of a fluorescent signal from the enzyme substrate if it contains exosomal immune complexes. By detecting the fluorescence intensity of these droplets, the concentration of the target sEVs can be deduced (shown in Fig. 5(A)). This device achieved a low detection limit of 10 enzyme-labeled sEVs complexes/µL, showing high sensitivity and specificity. This microfluidic biosensor is suited for early detection and dynamic monitoring of patients with early-stage breast cancer, as well as individuals within high-risk populations. Its detection limit of 10 sEVs/µL, combined with high sensitivity for capturing small sEVs at low concentrations, makes it ideal for frequent monitoring. This enables the provision of reliable biomarker data, even during the early stages of the disease. Therefore, the sensor is useful for early diagnosis and prognostic monitoring of breast cancer, which can contribute to timely intervention and personalized treatment strategies.

Another approach is the microfluidic magnetic sorting-based method. This utilizes microfluidic channel structures to design systems for sorting and manipulating immunomagnetic beads. An external magnetic field directs the flow of immunomagnetic beads, achieving selective capture and enrichment of target markers. Microfluidic magnetic sorting typically offers advantages of low sample usage, high sorting speed, automation, low cost, and reusability. Based on this, some researchers have recently utilized dimethyl dithiobispropionimidate (DTBP) as an immunomagnetic bead to achieve isolation of cfDNA from the plasma of colorectal cancer patients in a microfluidic platform [133]. The microfluidic channel structure guided the flow of immunomagnetic beads using an external magnetic field, enabling selective capture and enrichment. This device was both simple and cost-effective, providing rapid separation of cfDNA from plasma in just 15 min (shown in Fig. 5(B)). This microfluidic biosensor is suitable for patients at high risk of colorectal cancer, postoperative recurrence monitoring and efficacy assessment. Its test sample is plasma, which is easy and less invasive to collect, making it suitable for use in routine physical examinations and follow-up visits. In addition, the biosensor’s ability to rapidly isolate cfDNA in less than 15 min significantly improves diagnostic efficiency, and its detection of cfDNA as a marker allows researchers to analyze and provide precise information on tumor load and dynamics for personalized treatment and early intervention.

Furthermore, in recent studies, researchers have also directly integrated immunomagnetic beads into microfluidic devices, which is considered a cost-effective method for practical applications due to its low preparation cost, ease of large-scale production, and high degree of adjustability and reusability. For example, researchers developed a microfluidic biosensor combining individual magnetic bead capture and acoustic mixing techniques for the detection of PSA and CEA from serum [145]. The microfluidic biosensor used a permalloy (NiFe81/19) microarray to retain individual magnetic microbeads, forming a monolayer structure. After rapid mixing with the detection antibody, the magnetic beads were recaptured, and the image was analyzed using ImageJ and MATLAB Image Processing Toolbox to determine the marker concentration in the serum (shown in Fig. 5(C)).

Immunomagnetic bead-based microfluidic devices offer high sensitivity and selectivity, enabling rapid and accurate detection of target molecules in complex samples, conducive to early cancer diagnosis. The clinical significance of this technology lies in its ability to isolate and concentrate rare target molecules, such as CTCs and sEVs, enhancing the detection sensitivity. This approach is beneficial for liquid biopsies, offering a rapid, non-invasive method for cancer diagnosis, prognosis, and monitoring of treatment responses. However, they have certain drawbacks, such as the high cost associated with the fluorescent magnetic bead labeling technique, which requires specialized equipment and reagents. Additionally, the fluorescence signal’s susceptibility to environmental factors necessitates the need of preventing fluorescence quenching.

### Microfluidic biosensors combining microfluidics and enzyme-linked immunosorbent assays (ELISA)

**Fig. 6:**
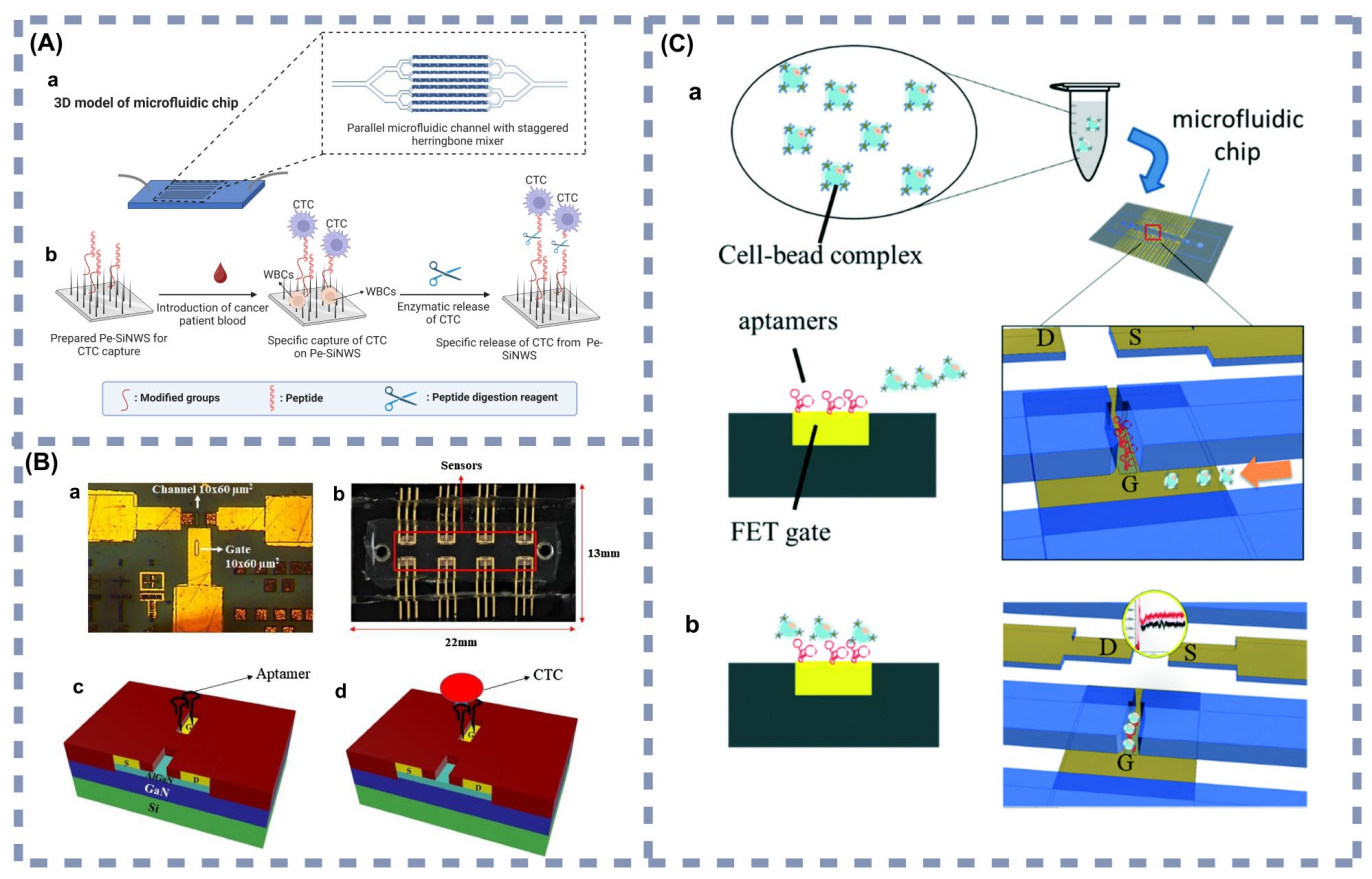
(**A**) Microfluidic devices combined with peptide-functionalized silicon nanowires. **a**: 3D model of microfluidic chip. **b**: Diagrammatic sketch of CTC capture on Pe-SiNWS and release from Pe-SiNWS. Reprinted with permission [127]. Copyright (2019), with permission from Dove Medical Press; (**B**) Microfluidic device based on AlGaN/GaN HEMT arrays. **a**: Top view image of 1 mm2 AlGaN/GaN HEMT device. **b**: 8-sensor array embedded on an epoxy substrate integrated with flow channel. **c**: Schematic of aptamer functionalized AlGaN/GaN HEMT sensor. **d**: Schematic representation of CTCs captured by aptamer immobilized on the gate opening of HEMT sensor. Reprinted with permission [128]. Copyright (2018), with permission from Elsevier; (**C**) A schematic illustration of the CTC capture and detection processes. a: sample injection; b: CTC trapping and FET sensing. D = drain, S = source, and G = gate. Reprinted with permission [129]. Copyright (2019), with permission from Royal Society of Chemistry. CTCs: Circulating tumor cells. Pe-SiNWS: Biological functionalization of silicon nanowire chip. PDMS: Polydimethylsiloxane. WBCs: White blood cells. HEMT: High electron mobility transistor. FET: field-effect transistors

ELISA is a technique leveraging the specific binding of antigens and antibodies to capture target molecules. Coupled with microfluidic technology, it achieves sample miniaturization and high-efficiency processing. Various forms exist for combining ELISA with microfluidic devices, with the prevalent type being enzyme-labeled microfluidic chips for early cancer detection. In this approach, enzyme-labeled antibodies or ligands immobilize on the microfluidic chip surface, facilitating the specific capture of tumor markers [135]. This approach is well embodied in a microfluidic biosensor for capturing and releasing CTC from the blood of pancreatic cancer patients [127]. The microfluidic biosensor featured a specialized microfluidic channel enhancing cell-substrate interactions, elevating CTCs capture efficiency. EpCAM from pancreatic cancer cells was specifically captured using CKAAKN (a peptide specific to pancreatic cancer cells). In post-capture, cells were released via enzymatic digestion to minimize damage and increase cell survival (shown in Fig. 6(A)). This microfluidic biosensor is suitable for early screening and treatment monitoring in individuals at high risk of pancreatic cancer (e.g., those with a family history or chronic pancreatitis), as well as those already diagnosed. The sample consists of single nucleated cells from peripheral blood, which can be collected non-invasively, though specialized equipment is required. The biosensor achieves a capture efficiency of 95.6%, a release efficiency of 92.6%, and maintains cell viability at 93.5%, highlighting its efficient and gentle handling of CTCs for isolation and analysis. As such, the sensor is suited for use in clinical settings, offering high-precision, real-time pathological data for personalized treatment of pancreatic cancer, thereby supporting improved prognosis management.

Another prevalent approach involves combining enzyme labeling with electrochemical detection. Here, tumor marker capture is achieved by immobilizing enzyme-labeled antibodies or ligands in a microfluidic system. Subsequent introduction of electrochemical detection methods, such as electrochemical sensors or microelectrodes, allows the detection of electrochemical signals associated with the enzyme-linked reaction. Based on this concept, Pulikkathodi and coworkers developed a microfluidic biosensor based on a GaN high electron mobility transistor (HEMT) biosensor array for capturing, detecting, and counting CTCs of colorectal cancer in blood [128]. The device utilized an AlGaN/GaN HEMT as a biosensor, employing aptamer as a receptor immobilized on the electrode. When CTCs flowed through the device and bind to the aptamer, a change in current gain was induced, thus achieving CTCs detection (shown in Fig. 6(B)). Similarly, Chen et al. [129] integrated a microfluidic chip and field-effect transistor (FET) sensor array for automated detection and counting of CTCs from serum of colorectal cancer patients. The device combined enzymatic labeling and electrochemical detection by immobilizing an enzyme-labeled CTC-specific antibody in the microfluidic system. The FET sensor detected electrochemical signals associated with the enzymatic reaction, quantifying the number of CTCs in the sample by the change in electrochemical signals (shown in Fig. 6(C)). This approach holds promise for early cancer detection through reduced sample processing errors and operator bias. The clinical potential of this type of microfluidic biosensor lies in its ability to perform multiplexed detection of various cancer biomarkers simultaneously, enabling comprehensive profiling of the patient’s cancer status. While the combination of microfluidic devices and ELISA achieves highly sensitive and selective detection of cancer markers, it demands more from samples and necessitates antibody preparation in advance. Issues may arise in accuracy if the target molecule concentration is too low. Therefore, future development of such microfluidic devices should focus on addressing these challenges to enhance overall performance.

Microfluidic biosensors, categorized based on their integration with electrochemical sensors, SERS, magnetic-based enrichment, and ELISA, each offer distinct clinical advantages. Electrochemical sensors provide precise and rapid quantification of low-abundance biomarkers, making them suitable for early detection and continuous monitoring. SERS-based biosensors offer detailed molecular insight, aiding in the identification and differentiation of cancer types for personalized treatment. Immunomagnetic bead-integrated systems excel in isolating and enriching rare cancer-related molecules, enhancing detection sensitivity, and enabling non-invasive liquid biopsies. ELISA-based microfluidic biosensors facilitate multiplexed detection, offering comprehensive cancer profiling for better diagnostic and prognostic outcomes. These technologies collectively enhance the accuracy, efficiency, and applicability of early cancer detection in clinical practice.

In addition to these major types, researchers in recent years have introduced various other microfluidic devices for early cancer detection, as detailed in Table 3.

In clinical environments, the application of these microfluidic biosensors may be explored through multiple pathways. For example, clinical trials may serve to validate their effectiveness in real-world settings by comparing their diagnostic performance with conventional methods. Integration into point-of-care testing platforms may enable rapid results in outpatient settings, thereby improving patient management and facilitating informed treatment decisions. Moreover, these biosensors may be employed in routine screening programs to monitor at-risk populations, supporting early intervention strategies. It is worth emphasizing that the collaboration of sensor developers with healthcare professionals and regulatory bodies will be essential to ensure that these technologies meet established safety and efficacy standards. Such collaborations will facilitate their adoption into clinical practice. Collectively, these technologies enhance the accuracy, efficiency, and applicability of early cancer detection, with the potential to transform clinical approaches to cancer diagnosis and management.

**Table 3:** Microfluidic devices developed in the last five years for early cancer detection

No	Mdf	Ct	Type	Markers	Dl/Ce	Principle	Ref
1	Combination of microfluidics and electrochemical immunosensors	Melanoma	Peripheral blood	MC1R	10 cells/10 mL	Covalently attached antibodies to the cell surface antigen MC1R are integrated onto a polyaniline nanofiber-modified working electrode surface on a screen-printed electrode.	[124]
2	Microfluidic device and LAMP technology combined	Lung cancer	Cancer tissue	CEA, CYFRA21-1, SCCA, NSE, ProGRP, CA125, EGFR, IDH1, TTF-1, SYN, CD56.	10 copies for NSE, ProGRP, and CD56. 10^2^ copies for the other biomarkers.	LAMP targeting 11 biomarkers and one internal reference are embedded on the microfluidic chip’s bio-reactor cells.	[125]
3	Microfluidic devices combined with photodynamic and immunocapture technologies	Prostate Cance	Urine	PSMA	10 cells/60 µL	The microfluidic device operates on a biocompatible polymeric film coated with anti-PSMA antibodies. This coating selectively binds prostate cancer cells in urine.	[126]
4	Microfluidic devices combined with peptide-functionalized silicon nanowires	Pancreatic Cancer	Blood	EpCAM	95.60%	Incorporate a staggered herringbone structure to enhance the capture efficiency of CTCs.	[127]
5	Microfluidic device based on AlGaN/GaN HEMT arrays	Colorectal Cancer	Blood	CTCs	-	The capture of CTCs on the gate electrode causes a change in the solution capacitance, which in turn modulates the transistor drain current, and measured by the HEMT sensor.	[128]
6	Combination of microfluidic devices and magnetic nanoparticles	Colorectal cancer	Blood	EpCAM, EGFR,HER-2, MUC-1	80%	Capture CTCs from blood by using antibody-modified magnetic immunoliposomes, and then CTCs are observed and counted under a fluorescence microscope.	[129]
7	Microfluidic devices with combined with quantum dot labeling and vesicle fusion technology	Pancreatic Cancer	Blood	CD81, EphA2, CA 19 − 9, miR-451a, miR-21, miR-10b	Protein: 16–28 exosome/µL. MiRNA: 14–22 exosome/µL	3D microfluidic chip is used with two functional channels, each modified with biotinylated gelatin film and streptavidin-linked polystyrene spheres.	[87]
8	Capillary-based microfluidic biosensing device	Breast Cancer	Cancer Cell Culture Solution	-	Single cell encapsulation efficiency of 38.8%	Capture circulating CTCs by encapsulating single cells in nanoliter compartments.	[130]
9	Microfluidic device combining nickel (Ni) micropillars and electrostatically spun PLGA nanofibers	Breast Cancer	Plasma and peripheral blood	EpCAM	8 cells/mL	PLGA nanofibers are stacked transversely on the surface of Ni microcolumns by electrostatic spinning to construct a three-dimensional biomimetic interface for trapping EpCAM-expressing CTCs.	[131]
10	Microfluidic device combining immunosorbent fluorescent magnetic beads	Breast Cancer	Serum	GPC-1	10 enzyme-labeled exosome complexes/µL	Exosomes are captured from serum using magnetic beads conjugated to CD63 antibody.	[132]
11	Microfluidic device based on DTBP	Colorectal cancer	Plasma and tissues	cfDNA	-	Surface of the thin film microfluidic platform is modified with DTBP, which binds to the amine groups of cfDNAs via electrostatic coupling and covalent bonding.	[133]
12	Microfluidic chip using Au nanoparticle–modified SiO_2_ microsphere (Au@SiO_2_) array–based highly active SERS substrates	Cervical cancer	Serum	SCCA, CEA	SCCA: 0.45 pg/mL, CEA: 0.36 pg/mL	SERS labels are produced using antibodies conjugated to Ag nanocubes. Intensity of the SERS signal correlates with the concentration of CEA versus SERS.	[134]
13	Microfluidic devices combining functionalized antibodies	Liver cancer	Blood	ASGPR and EpCAM	85%	Microcolumn array designed based on lateral displacement allows CTCs and blood cells to migrate through the fluid with different paths and different probabilities of collision with the microcolumns to isolate the CTCs.	[135]
14	Microfluidic device with lantern-like bypass structure combined with SERS	Hepatocellular carcinoma	Blood	TLS11a protein and VIM	84%	Utilize a microfluidic channel with a lantern-like bypass to capture CTCs from whole blood samples.	[136]
15	Microfluidic devices based on SERS and dielectrophoresis	Breast Cancer	Blood plasma	CTCs	20 cells/mL	Alternating electric field with specific parameters (e.g., frequency, voltage, and deposition time) is applied to a microfluidic channel containing a CTC sample.	[137]
16	Unique microfluidic disc system combining aptamer magnetic bead bioconjugate with a HRP-probe attached	Lung cancer	Blood	PD-L1, CA125, CD63, CEA, EpCAM	PD-L1: 1.58×10^5^ particles/mL	The intensity of the fluorescence signal is proportional to the concentration of the HRP-probe complex, which indirectly reflects the density of captured exosomes.	[138]
17	A detachable microfluidic device combining DeMEA and microfluidic vortexer	Breast Cancer	Plasma	EpCAM	17 exosomes/µL	The sensor surface was modified using nanocomposites to enhance its electrochemical properties.	[139]
18	Microfluidic device combining magnetic nanoparticles and in situ Raman assay	Prostate cancer	Serum	CD 63	1.6 × 10² particles/mL	Efficient exosome capture using anti-CD63 conjugated magnetic nanoparticles through mixing channels of a staggered triangular pillar array.	[140]
19	Microfluidic device combining immunocapture and SERS	Osteosarcoma	Plasma	CD63, EpCAM, VIM	2 particles/µL	AuNPs@MBA@anti-CD63, AuNPs@TFMBA@anti-VIM, and AuNPs@MPY@anti-EpCAM SERS tags were used to bind exosomes in plasma to form exosome immunocomplexes.	[141]
20	A novel SERS microfluidic device combining a catalytic hairpin assembly	Non-small cell lung cancer	Serum	ctDNA (TP53, PIK3CA-Q546K)	TP53: 2.26 aM, PIK3CA-Q546K: 2.34 aM	Two Raman signal molecules (4-MBA, DTNB) and two hairpin DNAs (HP1-1, HP1-2) were modified on Au–AgNSs to create SERS probes.	[142]
21	A novel SERS microfluidic device based on hpDNA-functional Au-AgNBs array	Non-small cell lung cancer	Serum	miR-92a and miR-339-3p	miR-92a: 42.14 aM, miR-339-3p: 56.37 aM	The reaction zone of the microchannel was covered with ordered Au-AgNBs arrays to capture miRNA molecules in the serum and to enhance SERS signals.	[143]
22	Triple-layer microfluidic device locally assembled from nanomaterials	Breast Cancer	Serum	miR-125, miR-126, miR-191, miR-155, miR-21	0.146 aM	FAM-labeled ssDNA served as DNA probes in the microchannel. DNA-miRNA complexes are formed, desorbed, and pushed into the assay chamber for collection and quantification.	[144]
23	A microfluidic device based on magnetic-based single bead trapping and acoustic micromixing	Prostate cancer	Plasma	PSA, CEA	PSA: 0.028 ng/mL, CEA: 3.1 ng/mL	A permalloy microarray in the device, flanked by two permanent magnets, facilitates the retention of magnetic beads for binding to exosomes.	[145]

No: Number

Mdf: Microfluidic device features

Ct: Cancer type

Dl/Ce: Detection limit/Capture efficiency

MC1R: Melanocortin 1 receptor

LAMP: Loopmediated isothermal amplification

ProGRP: Pro-gastrin-releasing peptide

EGFR: Epidermal growth factor receptor

IDH1: Dehydrogenase 1

TTF-1: Thyroid transcription factor-1

SYN: Synaptophysin

CD56: Neural cell adhesion molecule

PSMA: Prostate-Specific Membrane Antigen

EpCAM: Epithelial cell adhesion molecule

CTCs: Circulating tumor cells

HEMT: High electron mobility transistor

HER-2: Human epidermal growth factor receptor 2

MUC-1: Mucin1

EphA2: Ephrin type-A receptor 2

PLGA: Poly (lactic-co-glycolic acid)

DTBP: Dimethyl dithiobispropionimidate

SERS: Surface-Enhanced Raman Spectroscopy

ASGPR: Asialoglycoprotein receptor

VIM: Vimentin

PD-L1: Programmed cell death 1 ligand 1

HRP: Horseradish Peroxidase

DeMEA: Electrochemical aptasensor

MBA: Mercaptobenzoic acid

MPY: Mercaptopyridine

DTNB: 5,5’-dithiobis-2-nitrobenzoic acid

Au-AgNBs: Au-Ag nanobowl

FAM: 6-carboxyfluorescein

## Conclusions and future perspectives

This review provides a comprehensive review of the fabrication methods, materials, and biomarkers associated with microfluidic devices used in early cancer detection, aiming to provide general readers with a better understanding of this field. Subsequently, we highlight the significant advantages of microfluidic-based biosensors in detecting clinically relevant cancer biomarkers particularly for early diagnosis, risk assessment, and real-time monitoring of cancer progression. Various types of microfluidic biosensors are introduced to demonstrate their capability in improving the rapidity, sensitivity, and selectivity of detecting cancer biomarkers such as circulating tumor cells, extracellular vesicles, nucleic acids, protein biomarkers etc., by incorporating diverse nanomaterials and integrating multiple advanced detection technologies. Compared to traditional methods, microfluidic biosensors offer simplicity in operation with reduced processing time and eliminate the need for expensive analytical instruments, thus making them adaptable to point-of-care testing in clinical settings. Consequently, these biosensors hold great potential in liquid biopsy applications while providing a novel strategy for early detection and treatment of cancer. Furthermore, through precise and timely detection capabilities offered by microfluidic biosensors can help prevent unnecessary treatments and reduce the risk of drug overdose, thereby alleviating healthcare burdens on patients while conserving valuable healthcare resources.

Microfluidic devices play a pivotal role in healthcare resource management, allowing more strategic allocation based on predictive analytics for disease prevalence. Future trends in microfluidic device development could be the integration of multiple assays to enable simultaneous detection of diverse cancer biomarkers. Efforts should be paid to device miniaturization and portability to enhance the versatility of microfluidic devices for real-time diagnosis in various field applications. With recent advancements in artificial intelligence (AI), an emerging area of research lies in the integration of AI into microfluidic biosensors for intelligent, personalized data analysis and visualization.

Researchers are encouraged to focus on these aspects to develop microfluidic devices tailored for non-invasive cancer monitoring and treatment evaluation. Combined with the evolving needs of precision medicine, these new devices are promising to provide innovative solutions for early clinical cancer diagnosis.

## Data Availability

No datasets were generated or analysed during the current study.

## Abbreviations

PCR: Polymerase chain reaction
ddPCR: Droplet digital PCR
NGS: Next-generation sequencing
cfDNA: Circulating free DNA
ctDNA: Circulating tumor DNAs
TPP: Two-photon polymerization
PSA: Prostate-specific Antigen
CTCs: Circulating tumor cells
CEA: Carcinoembryonic antigen
AFP: Alpha-fetoprotein
HCG: Chorionic gonadotropin
CA19-9: Carbohydrate antigen 19 − 9
CA12-5: Carbohydrate antigen 12 − 5
CA72-4: Carbohydrate antigen 72 − 4
CA24-2: Carbohydrate antigen 24 − 2
CA15-3: Carbohydrate antigen 15 − 3
CYFRA21-1: Cytokeratin 19 fragment
NSE: Neuron-specific enolase
SCC: Squamous cell carcinoma-associated antigen
miRNAs: MicroRNAs
MC1R: Melanocortin 1 receptor
PANI: Polyaniline
SPE: Screen-printed electrode
PBMCs: Peripheral blood mononuclear cells
EpCAM: Epithelial cell adhesion molecule
AgNPs: Nanoparticles
SERS: Surface-Enhanced Raman Spectroscopy
LSPR: Localized surface plasmon resonance
AgNCs: Ag nanocubes
SCCA: Squamous cell carcinoma antigen
GPC-1: Glypican-1
DTBP: Dithiobispropionimidate
cfDNA: Circulating free DNA
ELISA: Enzyme-linked immunosorbent assays
HEMT: High electron mobility transistor
FET: Field-effect transistor
AI: Artificial intelligence
